# An epigenetic and transcriptomic signature of immune tolerance in human monocytes through multi-omics integration

**DOI:** 10.1186/s13073-021-00948-1

**Published:** 2021-08-16

**Authors:** Xanthe Brands, Bastiaan W. Haak, Augustijn M. Klarenbeek, Joe Butler, Fabrice Uhel, Wanhai Qin, Natasja A. Otto, Marja E. Jakobs, Daniël R. Faber, René Lutter, W. Joost Wiersinga, Tom van der Poll, Brendon P. Scicluna

**Affiliations:** 1 Center for Experimental Molecular Medicine, Amsterdam University Medical Centers, Academic Medical Center, University of Amsterdam, Amsterdam, 1105 AZ the Netherlands; 2 Laboratory of Genome Analysis, Amsterdam University Medical Centers, Academic Medical Center, University of Amsterdam, Amsterdam, 1105 AZ the Netherlands; 3 BovenIJ hospital, Amsterdam, 1034 CS the Netherlands; 4 Respiratory Medicine and Experimental Immunology, Amsterdam University Medical Centers, Academic Medical Center, University of Amsterdam, Amsterdam, 1105 AZ the Netherlands; 5 Division of Infectious Diseases, Amsterdam University Medical Centers, Academic Medical Center, University of Amsterdam, Amsterdam, 1105 AZ the Netherlands; 6 Department of Applied Biomedical Sciences, Faculty of Health Sciences, Mater Dei Hospital, University of Malta, Msida, Malta; 7 Centre for Molecular Medicine and Biobanking, University of Malta, Msida, Malta

**Keywords:** Monocytes, Pneumonia, Immune tolerance, Cytokines, DNA methylation, Epigenetics, Infection, Functional genomics

## Abstract

**Background:**

The plasticity of monocytes enables them to exert multiple roles during an immune response, including promoting immune tolerance. How monocytes alter their functions to convey immune tolerance in the context of lower respiratory tract infections in humans is not well understood. Here, we sought to identify epigenetic and transcriptomic features of cytokine production capacity in circulating monocytes during community-acquired pneumonia (CAP).

**Methods:**

Circulating CD14+ monocytes were obtained from the blood of CAP patients included in a longitudinal, observational cohort study, on hospitalization (acute stage, *n*=75), and from the same patients after a 1-month follow-up (recovery stage, *n*=56). Age and sex-matched non-infectious participants were included as controls (*n*=41). Ex vivo cytokine production after lipopolysaccharide (LPS) exposure was assessed by multiplex assay. Transcriptomes of circulating monocytes were generated by RNA-sequencing, and DNA methylation levels in the same monocytes were measured by reduced representation bisulfite sequencing. Data were integrated by fitting projection-to-latent-structure models, and signatures derived by partial least squares discrimination.

**Results:**

Monocytes captured during the acute stage exhibited impaired TNF, IL-1β, IL-6, and IL-10 production after ex vivo stimulation with LPS, relative to controls. IL-6 production was not resolved in recovery monocytes. Multivariate analysis of RNA-sequencing data identified 2938 significantly altered RNA transcripts in acute-stage monocytes (fold expression ≤−1.5 or ≥1.5; adjusted *p* ≤ 0.01), relative to controls. Comparing DNA methylation levels in circulating monocytes of CAP patients to controls revealed minimal differences, specifically in DNAse hypersensitive sites (HS) of acute-stage monocytes. Data integration identified a cholesterol biosynthesis gene signature and DNAse HS axis of IL-1β and IL-10 production (*R*^2^ =0.51).

**Conclusions:**

Circulating monocytes obtained from CAP patients during the acute stage exhibited impaired cytokine production capacities, indicative of reprogramming to a state of immune tolerance, which was not fully resolved after 1 month. Our split-sample study showed that 51% of the immune tolerance phenotype can be explained, at least in part, by coordinated shifts in cholesterol biosynthesis gene expression and DNAse HS methylation levels. A multi-scale model identified an epigenetic and transcriptomic signature of immune tolerance in monocytes, with implications for future interventions in immunosuppression.

**Trial registration:**

NCT number NCT02928367

**Supplementary Information:**

The online version contains supplementary material available at 10.1186/s13073-021-00948-1.

## Background

Monocytes are crucial orchestrators of the immune response. Long-thought of as a common source of tissue macrophages and dendritic cells [1, 2], mounting evidence points to more complex effector functions of monocytes. The plasticity of these cells enables them to exert multiple roles during an immune response, including pathogen recognition, cytokine release, phagocytosis, antigen presentation, and promoting immune tolerance [3, 4]. In the latter phenomenon, cells are reprogrammed to a state of transient refractoriness to secondary encounters with various antigens, including microbes or microbial products such as lipopolysaccharide (LPS) [5, 6]. Recent work also indicates that monocytes can develop a long-term memory subsequent to stimulation with pathogen moieties or vaccinations in a process termed “trained immunity” [7, 8]. Although extensive work has been done in dissecting the cellular pathways regulating murine monocyte plasticity and function, how circulating monocytes alter their functions in the context of lower respiratory tract infection in humans is less well understood.

Functional reprogramming of cells is driven by synchronized regulation of gene expression, which is achieved in large part by covalent modifications to chromatin, including methylation of the fifth carbon in cytosines (5-methylcytosine (5mC)) or DNA methylation, via epigenetic modulators [9, 10]. This level of coordination was shown in murine macrophages wherein Toll-like receptor (TLR) induced chromatin modifications were associated with transcriptional silencing of pro-inflammatory genes concomitant with activation of antimicrobial genes [11]. The importance of chromatin modifications to innate immune cell function is exemplified by influenza, which was shown to sabotage antiviral transcriptional responses by producing NS1 proteins that mimic histone tails and sequester epigenetic modulators [12, 13]. Recently, various products of immune cell metabolism, for example, mevalonate in the cholesterol biosynthesis pathway, fumarate accumulation in glutaminolysis, and itaconate, an anti-inflammatory product of the Krebs cycle, were also shown to influence innate immune cell activation and phenotype via chromatin modifications [14–16].

Here, we sought to broaden our understanding of monocyte functions captured during the acute and recovery stages of patients diagnosed with community-acquired pneumonia (CAP). By combining information on the cytokine production capacity upon LPS stimulation, RNA expression, and DNA methylation in a multi-omics model, we dissect the molecular characteristics underlying immune tolerance in circulatory monocytes.

## Methods

### Study population

In this longitudinal, observational cohort study, CAP patients (> 18 years) were included between October 2016, and June 2018, at the Internal Medicine ward or Intensive Care Unit of the Academic Medical Center and BovenIJ Hospital (Amsterdam, the Netherlands) within 16 h of hospital admission and revisited after 1 month. Patients were screened by trained research physicians and included when they were admitted with a primary suspicion of an acute infection of the respiratory tract, defined as reported fever or chills; documented fever or hypothermia, leukocytosis or leukopenia, new cough or sputum production, chest pain, dyspnea, tachypnea, abnormal lung examination, or respiratory failure; and had evident new or progressive infiltrate, consolidation, cavitation, or pleural effusion on a chest X-ray or computed tomography (CT) scan. Patients were excluded if there was a documented clinical suspicion of aspiration pneumonia or hospital-acquired pneumonia, or if patients were previously diagnosed with malignant hematological disease or exposed to chemotherapy, systemic corticosteroids, and/or other immunosuppressive drugs. In addition, patients were excluded if exposed to systemic antibiotics within 48 h prior to hospital admission. Age- and sex-matched subjects without acute infection were included as controls. Heparinized whole blood was obtained from all CAP patients and control participants for monocyte isolation, with purities >80% constituting an additional study inclusion criterion. In doing so, monocytes from 75 CAP patients on hospitalization (acute stage) and from the same patients after 1 month (*n*=56, recovery stage), as well as 41 control participants were included in the study. For DNA methylation analysis by reduced representation bisulfite sequencing (RRBS), the first subset of CAP patients as well as age, sex, and comorbidity-matched control participants were selected (CAP acute stage, *n*=26; CAP recovery stage, *n*=24; controls, *n*=22). To test the robustness of DNA methylation findings, the second subset of CAP patients and matched control participants were selected (CAP acute stage, *n*=21; controls, *n*=16). Trial registration number NCT02928367 (https://clinicaltrials.gov/ct2/show/NCT02928367).

### Monocyte isolation and purity

The heparinized blood was diluted (1:1) with phosphate-buffered saline (PBS). The peripheral blood mononuclear cells (PBMCs) were isolated by density-gradient centrifugation (1700 RPM for 30 min at 21°C, acceleration 1, breaks 0) using Ficoll-Paque PLUS (GE healthcare, 17-1440-02) and washed twice with cold PBS (GE Healthcare #M090001/02), supplemented with 0.5% sterile endotoxin-free bovine serum albumin (BSA, Divbio Science Europe, AK8917-0100). PMBCs were resuspended in MACS buffer (PBS having endotoxin-free 0.5% BSA, 2mM ethylenediaminetetraacetic acid (EDTA; Thermo-Fischer #AM9625)), containing magnetic beads coated with anti-CD14 antibodies (Miltenyi Biotec, 130-050-201), and incubated for 15 min on a roller bank kept at 4°C. CD14+ monocytes were purified using a LS MACS column (Miltenyi Biotec, QuadroMACS) and magnetic separator (Miltenyi Biotec). For the next-generation sequencing, 1 × 10^6^ purified monocytes were resuspended in 350ul RNAprotect cell reagent (Qiagen, Cat. #: 76526), and stored at −80^o^C. For ex vivo stimulations, purified monocytes were seeded in a cell-repellent surface 48-well plate (0.5×10^6^ cells per well) and incubated for 24 h at 37°C with 5% CO_2_ and 95% humidity in total 400μl Roswell Park Memorial Institute (RPMI; GIBCO, 31870-025) medium supplemented with 10% sterile fetal calf serum, 200mM glutamax (Thermo Fisher, 35050-038), 100 μM pyruvate (Thermo Fisher, 11360-039), and 50 μg/ml gentamycin (Lonza, 17-519Z), with or without 100ng/mL LPS (*Escherichia coli* 0111:B4 Ultrapure, Invivogen, Toulouse, France). Supernatants were stored at −80^o^C until analysis.

### Monocyte purity and subset determination

Fresh harvested PBMCs and monocytes were seeded in a polypropylene 96-well plate (0.2×10^6^ cells per well) and washed twice with flow cytometry staining buffer (PBS containing 0.5% endotoxin-free BSA, 2mM EDTA, and 0.1% NaN_3_ (Merck Millipore, 1687)). Cells were incubated with a mix of cell-specific antibodies (phycoerythrin anti-CD3 antibody (Ebioscience), fluorescein isothiocyanate-conjugated anti-CD66b, allophycocyanin anti-CD14, phycoerythrin cyanin-7 anti-CD56, and Alexa Fluor 700 anti-CD16 antibodies (BD Biosciences)), for 30 min at 4°C in the dark. Before analysis by FACS, cells were washed three times as described above and resuspended in a total volume of 300 μl FACS buffer. Monocyte purity was verified via flow cytometry (FACS Canto II with FACSDiva Software; BD Biosciences, Heidelberg, Germany).

### Cytokine measurements

TNF-α, IL-1β, IL-6, and IL-10 levels in supernatants were measured using a Luminex multiplex assay (R&D Systems Inc., Minneapolis, MN) and BioPlex 200 (BioRad, Hercules, CA).

### DNA and RNA isolation

The total RNA and genomic DNA were isolated from the same monocyte sample using the AllPrep DNA/RNA mini kit according to the manufacturer’s instructions (Qiagen). The RNA quality was assessed by bioanalysis (Agilent), with all samples having RNA integrity numbers > 9. The total RNA and genomic DNA concentrations were determined by Qubit® 2.0 Fluorometer (Life Technologies, Carlsbad, CA, USA).

### RNA sequencing

RNA-sequencing libraries were prepared from 200ng total RNA using KAPA RNA Hyperprep with RiboErase (Roche) library kits. Libraries were sequenced using the Illumina HiSeq4000 instrument (Illumina) to generate single reads (50bp). The sequencing depth was approximately 40 million reads per sample. Sequence read quality was assessed by means of the FastQC method (v0.11.5; http://www.bioinformatics.babraham.ac.uk/projects/fastqc/). After RNA-seq quality assessment, six CAP patient samples were flagged as poor quality and removed from further analysis. Trimmomatic version 0.36 [17] was used to trim Illumina adapters and poor-quality bases (trimmomatic parameters: leading=3, trailing=3, sliding window=4:15, minimum length=40). The remaining high-quality reads were used to align against the Genome Reference Consortium human genome build 38 (GRCh38) [18]. Mapping was performed by HISAT2 version 2.1.0 [19] with parameters as default. Count data were generated by means of the HTSeq method [20] and analyzed using the DESeq2 method [21]. Statistically significant differences were defined by Benjamini & Hochberg adjusted probabilities < 0.01 and absolute fold expression <−1.5 or >1.5. Canonical signaling pathways and biofunctions were generated by Ingenuity Pathway Analysis (QIAGEN bioinformatics) specifying human species and ingenuity database as reference. Benjamini & Hochberg adjusted probabilities < 0.05 demarcated significance. Deconvolution of absolute immune signal of monocyte transcriptomes was done by applying the online Shiny application (https://github.com/giannimonaco/ABIS) [22].

### Reduced representation bisulfite sequencing

RRBS libraries were prepared from 100ng of monocyte DNA using the Diagenode® Premium Reduced Representation Bisulfite Sequencing kit (Diagenode Europe, Belgium). Briefly, DNA was digested with the restriction enzyme MspI, which recognizes CCGG dinucleotide sites, and AMPpure XP kit (Beckman Coulter) for size selection. Samples were pooled based on concentrations (*n*=8 per pool) and treated with sodium bisulfite to convert the unmethylated cytosines to uracil leaving unmethylated cytosines unchanged. After bisulfite conversion, samples were amplified by PCR using the following reaction setup: 20 min at 25°C, 10 min at 65°C, hold at 8°C. PCR products were cleaned using the AMPpure XP kit and sequenced on the Illumina HiSeq4000 (Illumina) instrument in single read (50) mode.

### Reduced representation bisulfite sequencing data analysis

Sequence read quality was assessed by means of the FastQC method (v0.11.5; http://www.bioinformatics.babraham.ac.uk/projects/fastqc/). Sequences were mapped to the GRCh38 genome build using the bs-seeker2 method [23] and bowtie2 aligner [24] specifying the following parameters: lower bound-upper bound = 20–500, cut site pattern = C-CGG, tag = undirectional, mismatches allowed = 4, and bowtie2 = end-to-end. Methylation levels (number of C reads/[number of C + number of T]) were calculated using bs-seeker2 specifying parameters that remove reads which would be considered as not fully converted by bisulfite treatment. Methylation levels were summarized across exons, introns, CpG islands (CGIs), or DNAse hypersensitive sites (HS) using cgmaptools [25]. Genomic regions containing more than 5% missing values across all samples were removed. Data were logit transformed and used for statistical analysis using limma [26] by fitting a multivariate linear model that included age, sex, and sequencing library pool as covariates. Absolute methylation levels were used for graphical representation of results.

### Data integration

To build a multi-omics model of monocyte function in CAP, we used data integration analysis for biomarker discovery using latent components (DIABLO) [27], in the R library mixOmics [28]. Using complete cases that overlap the three data blocks (delta cytokines, RNA-seq, DNA-meth), and considering delta cytokines block contained four variables, we calculated the first 4 projection to latent structure (PLS) components. Top most informative variables per block were identified by, firstly using loading vectors per PLS component to select those variables in the upper or lower 20% of the distribution. Secondly, classification by sparse partial least squares regression and LASSO L1 penalization. Classification performance was assessed by repeated (10X) 5-fold cross-validation and receiver-operator-characteristic area-under-the-curves. Correlations were calculated using Pearson’s method, and the effect size was estimated by calculating coefficients of determination (*R*^2^).

### Methylated DNA immunoprecipitation (MeDIP)

MeDIP analysis was performed using Methylamp Methylated DNA Capture Kit (Epigentek, Farmingdale, NY) following the manufacturer’s instructions. For this analysis, we obtained monocyte DNA from a subset of 21 CAP patients during acute disease stages, and 16 control participants. Briefly, genomic DNA was fragmented by sonication using a Diagnode BioRuptor (Diagenode). In total, 100 ng of fragmented DNA was added to every 5-mC antibody-coated well and incubated at room temperature on a horizontal shaker for 2 h. Immunoprecipitated DNA was released by proteinase K and eluted in 100 μl nuclease-free water. We calculated 5-mC levels in immunoprecipitated DNA relative to input DNA at the DNAse HSs located in chromosome 22 (39,063,900bp-39,064,310bp; DNAse-HS-Chr22) and chr8 (143,985,240bp-143,985,390bp; DNAse-HS-Chr8) using the 2^ΔCt method. The following primer pairs were used:

DNAse-HS-Chr22, Forward primer - 5′: GAACCAGAGGTGCCAGAGAA

DNAse-HS-Chr22, Reverse primer - 5′: TTGGGCACATGTTATCAGGA

DNAse-HS-Chr8, Forward primer - 5′: GGCTCCAGGATGTCGTAGTG

DNAse-HS-Chr8, Reverse primer - 5′: TCCTTTCCCTTCCTCCAAGT

### Publicly available data analysis

DNA methylation data from sepsis patients and healthy controls were obtained from GEO with accession number (GSE138074) [29]. Data were generated using Infinium MethylationEPIC BeadChips (Illumina, Inc. San Diego, CA, USA). Despite the beadchips having methylation sites that are incompletely ascertained (representing ~2% of CpGs and ~99% of RefSeq genes enriched in promoters and genic bodies), we used the available processed dataset to select probes based on the GRCh37 genome build that overlapped with regions of interest as delineated by our RRBS analysis. Patients with sepsis due to “respiratory” diagnosis (*n*=4) and healthy controls matched for age and sex (*n*=6) were used. Promoter capture Hi-C interactions, specifically for monocytes and macrophages, were downloaded from previously described Hi-C data [30].

### Clinical variables

Vital parameters and severity scores, such as the quick Sequential Organ Failure Assessment (q)SOFA score [31] and Pneumonia Severity Index (PSI) [32], were calculated on hospital admission. Immunosuppression was defined by use of methotrexate or prednisone, and/or positive human immunodeficiency virus.

### Statistical analysis

Statistical analysis was performed in the R statistical framework (Version 3.51, R Core Team 2014. R: A language and environment for statistical computing. R Foundation for Statistical Computing, Vienna, Austria). All results are presented as numbers (percentages) for categorical variables, median, and quartiles (Q1-Q3) for nonparametric quantitative variables, and mean ± standard deviation of the mean (SD) for parametric quantitative variables. Data distribution was assessed by the Kolmogorov-Smirnov test. Continuous nonparametric data were analyzed using a Mann-Whitney *U* test or a Kruskal-Wallis with Dunn’s post hoc test; categorical data were analysed using a χ2 or Fisher’s exact test. Continuous parametric data were analyzed using a Student *t* test or analysis of variance. A *p* value < 0.05 was considered statistically significant. Multiple-comparison adjusted (Benjamini-Hochberg) *P* value less than 0.05 defined significance of plasma biomarker results [33]. Boxplots were generated using ggplot2 [34].

A comprehensive list of reagents, resources, and computational methods can be found in Additional file 1.

## Results

### Cytokine production of stimulated monocytes is partially impaired and resolved during pneumonia

**Fig. 1:**
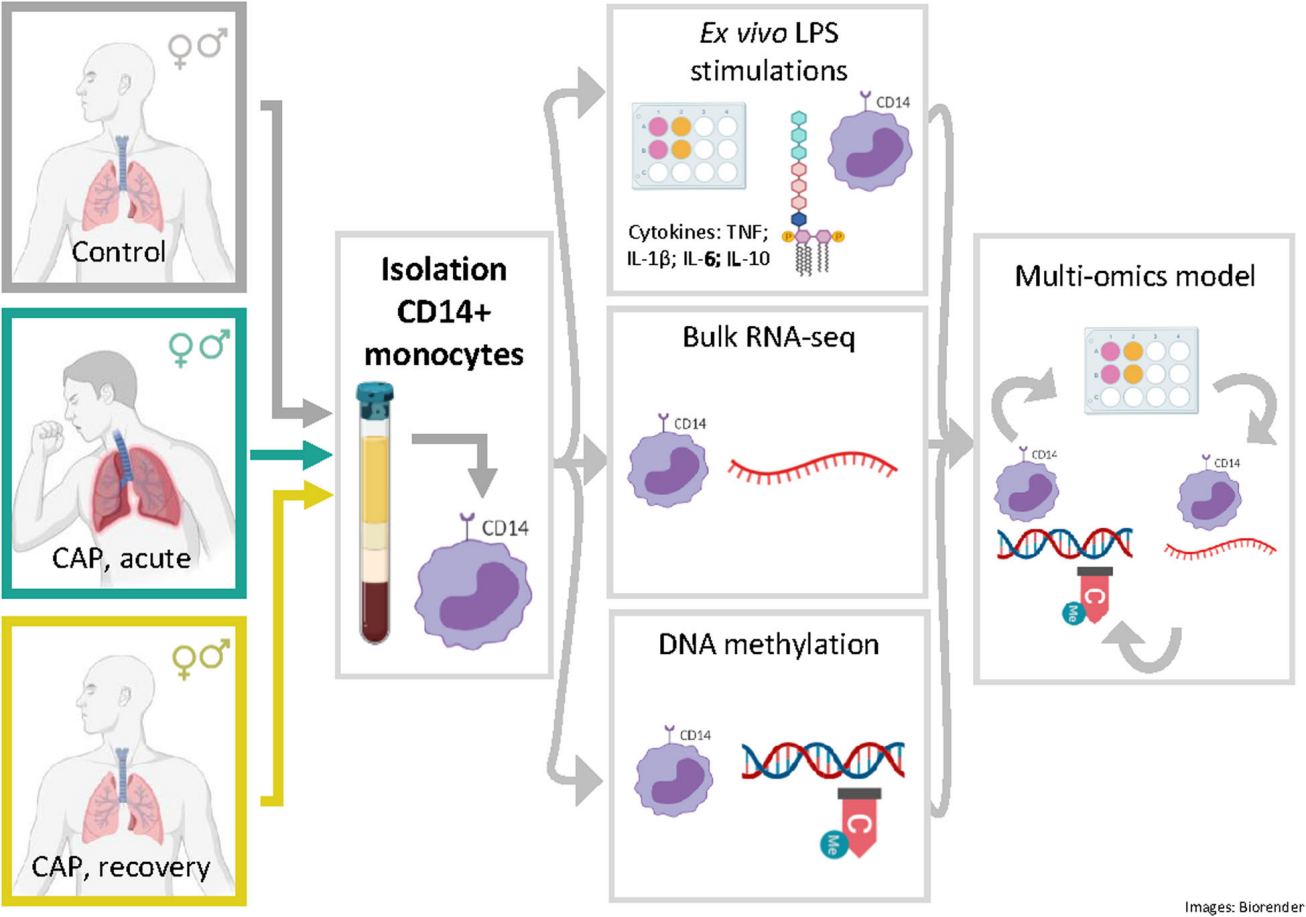
Schematic representation of study workflow. Purified CD14-positive (+) monocytes (CD14+ purities ≥ 80%) obtained from community-acquired pneumonia (CAP) patients on study inclusion (hospital admission; acute stage, *n*=75), from the same patients after 1 month of study inclusion (recovery stage, *n*=56), as well as age and sex-matched participants without acute infection (controls, *n*=41). Monocytes were stimulated for 24 h with *Escherichia coli* lipopolysaccharide (LPS). CAP community-acquired pneumonia

**Fig. 2:**
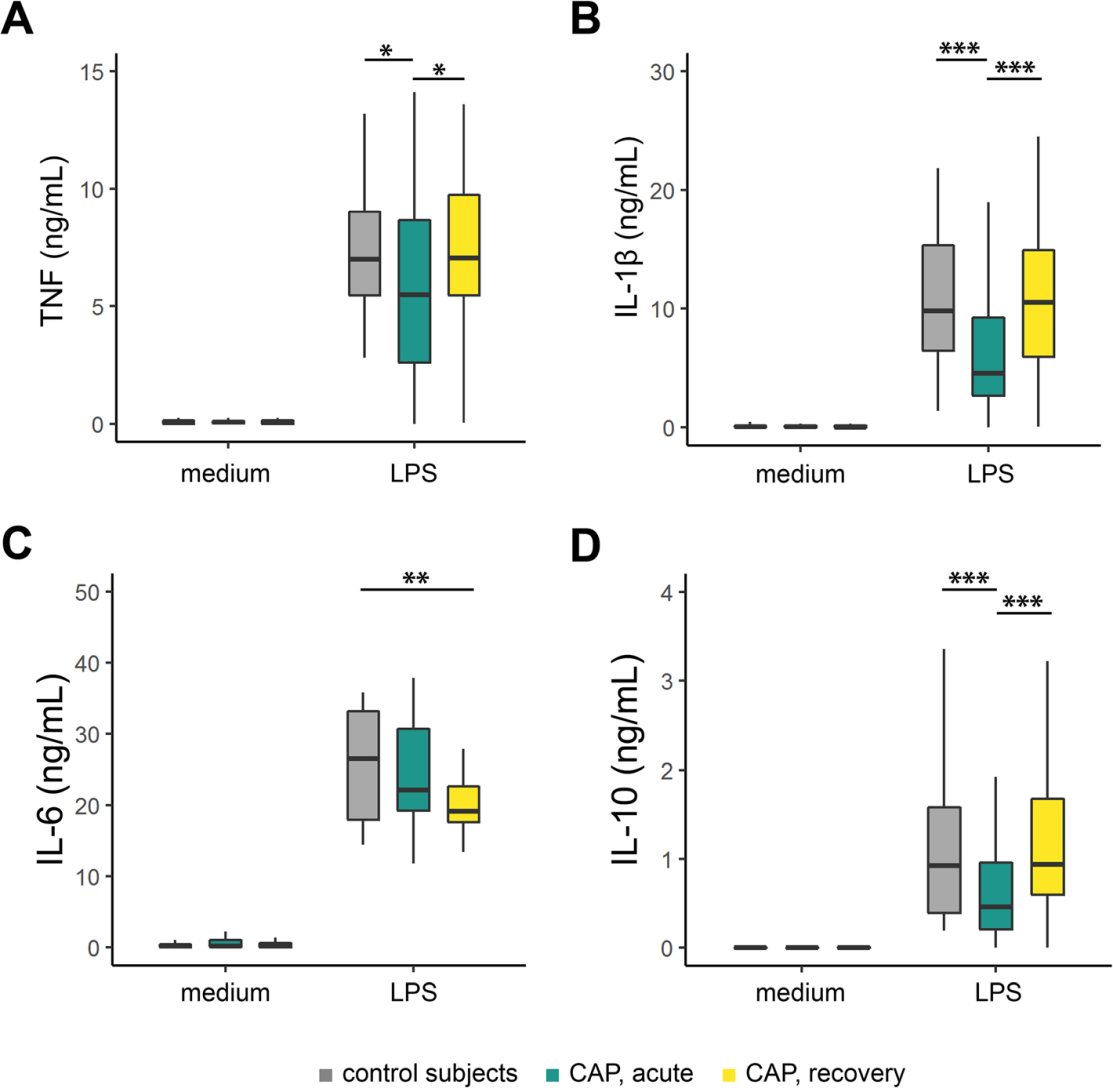
Ex vivo cytokine production capacity of circulating monocytes from patients with community-acquired pneumonia in the acute and recovery stage. Boxplots depicting **A** tumor necrosis factor (TNF), **B** interleukin (IL-)1β, **C** IL-6, and **D** IL-10 production by circulating monocytes in response to stimulation with *Escherichia coli* lipopolysaccharide (LPS, 100 ng/mL) for 24 h. Data are expressed as box and whisker diagrams depicting the median and lower quartile, upper quartile, and their respective 1.5 interquartile range as whiskers (as specified by Tukey). *Kruskal-Wallis Dunn’s post hoc test *p* < 0.05, **p < 0.01, ***p < 0.001

In order to determine cytokine production capacities of circulating monocytes during pulmonary infection, we stimulated purified CD14-positive (+) monocytes (CD14+ purities ≥80%; Additional file 2: Fig. S1A, S1B), with *Escherichia coli* LPS for 24 h, obtained from CAP patients on hospital admission (acute stage, *n*=75), from the same patients after 1 month follow-up (recovery stage, *n*=56), as well as age and sex-matched participants without acute infection (controls, *n*=41) (Fig. 1). By applying a deconvolution of absolute immune signal method [22] to CD14+ monocyte transcriptomes, we found robust enrichment of classical monocytes, and to a lower extent of non-classical monocytes (Additional file 2: Fig. S1C). FACS analysis of CD14 and CD16 cell-surface markers, which can classify classical, non-classical and intermediate monocyte subsets [35, 36], revealed monocytes obtained from control participants and CAP patients were highly enriched for CD14^pos^/CD16^neg^ classical monocytes. Of note, monocytes of CAP patients captured during acute disease stages were characterized by a significant decrease in classical monocytes (CD14^pos^/CD16^neg^), concomitant with expansion of intermediate (CD14^pos^/CD16^pos^) and non-classical (CD14^dim^/CD16^pos^) monocyte subsets, relative to controls or CAP recovery monocytes (Additional file 2: Fig. S1D). Characteristics of patients and control participants are tabulated in Table 1. Patients and controls were similar in demographics, but patients had higher counts of chronic obstructive pulmonary disease (COPD) and diabetes. *Streptococcus pneumoniae*, *Haemophilus influenzae*, and Influenza (H1N1/H3N2) were the predominant causal pathogens detected (Table 1). Robust cytokine responses were observed after stimulating monocytes obtained from controls and CAP patients during acute or recovery stages (Fig. 2). Tumor necrosis factor (TNF), interleukin (IL-)1β, IL-6, and IL-10 were significantly induced after LPS stimulation relative to medium. After LPS stimulation, production of TNF, IL-1β, and IL-10 by acute stage monocytes were significantly reduced relative to controls, but not for IL-6 (Fig. 2A-D). While the production of TNF, IL-1β, and IL-10, obtained after 1 month of CAP patients’ hospital admission (recovery monocytes) were restored to those of control levels, IL-6 production was significantly reduced. These observations indicate that circulating monocytes obtained from CAP patients during the acute stage of the disease have predominantly reduced cytokine production capacities, indicative of immune tolerance, which are not fully resolved after one-month recovery.

**Table 1:** Baseline characteristics of community-acquired pneumonia patients and control participants

	CAP patients	Control subjects	***P*** value
Size, *n*	75	41	
**Demographics**			
Age, year, mean (SD)	70.67 (13.31)	69.37 (8.60)	0.574^d^
Sex, male, *n* (%)	41 (54.7)	24 (58.5)	0.837^c^
Ethnicity, Caucasian, *n* (%)	53 (70.7)	36 (87.8)	0.063^c^
Body Mass Index, median [Q1, Q3]	25.93 [23.07, 28.41]	26.36 [24.74, 29.58]	0.177^b^
**Chronic comorbidities,*n*(%)**			
COPD	26 (34.7)	4 (9.8)	0.007^c^
Cardiovascular disease	58 (77.3)	25 (61.0)	0.099^c^
Diabetes	19 (25.3)	4 (9.8)	0.077^c^
Malignancy	20 (26.7)	8 (19.5)	0.526^c^
Chronic renal disease	7 (9.3)	1 (2.4)	0.309^c^
Immunosuppression^a^	4 (5.3)	2 (4.9)	0.99^c^
**Disease severity, median [Q1, Q3]**			
PSI score	4.00 [3.00, 4.00]	–	
SOFA score	0.00 [0.00, 1.00]	–	
**Causal pathogen,*n*(%)**		–	
*Streptococcus pneumoniae*	10 (13.33)	–	
*Haemophilus influenzae*	7 (9.33)	–	
*Staphylococcus aureus*	4 (5.33)	–	
Influenza A virus	6 (8)	–	
Influenza B virus	3 (4)	–	
Rhinovirus	3 (4)	–	
Coronavirus type NL63	1 (1.33)	–	
Respiratory syncytial virus	2 (2.67)	–	
Human metapneumovirus	2 (2.67)	–	
Parainfluenza virus 1–4	2 (2.67)	–	
No causative pathogen identified	39 (52)	–	
**Outcome**			
ICU admission, *n* (%)	5 (6.7)	–	
Hospital LoS, days, median [Q1, Q3]	5.00 [3.00, 8.75]	–	
Hospital mortality, *n* (%)	1 (1.4)	–	
Day 28 mortality, *n* (%)	4 (6.1)	–	

Definition of abbreviations: *CAP* community-acquired pneumonia, *COPD* chronic obstructive pulmonary disease, *PSI* Pneumonia Severity Index, *SOFA* Sequential Organ Failure Assessment, *IC*U intensive care unit, *LoS* length of stay, and *SD* standard deviation of the mean. ^a^ Immunosuppression was defined by the use of methotrexate or prednisone (*n*=2) and/or positive human immunodeficiency virus (*n*=5). ^b^ Mann-Whitney *U* test; ^c^ chi-square test with Yates’ continuity correction; ^d^ Student *t* test

### Elevated inflammatory and antibacterial pathway concomitant with reduced protein translation and antigen presentation characterize acute stage monocytes

**Fig. 3:**
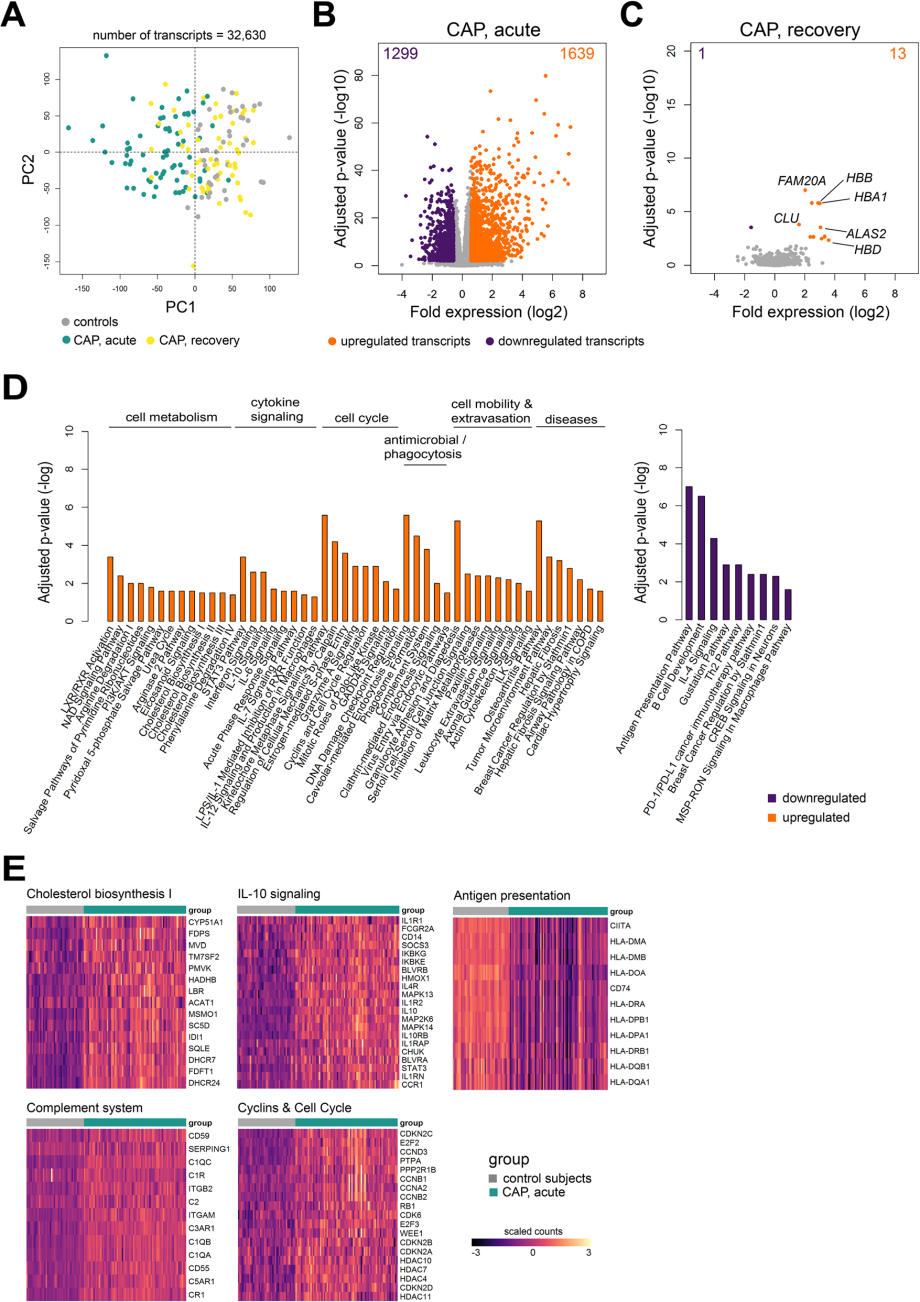
Transcriptomic profiling of circulating monocytes in the acute and recovery stage of community-acquired pneumonia. **A** Principal component (PC) plot depicting PC1 and PC2 of 32,630 transcripts of monocytes sampled from CAP patients (acute, *n* = 75; recovery, *n* = 59) and control subjects (*n* = 41). **B**, **C** Volcano plot representation (integrating log2 fold change and multiple-comparison adjusted *p* values) of genome-wide alterations in RNA expression of CAP patient monocytes obtained during (**B**) the acute stage, and (**C**) recovery stage, relative to control monocytes. *upregulated genes, absolute fold expression >1.5 and adjusted *p*<0.01; *downregulated genes, absolute fold expression ≤1.5 and adjusted *p*<0.01. **D** Bar plots illustrating significantly enriched ingenuity’s canonical signaling pathways in acute stage monocytes. **E** Heatmaps of upregulated genes and downregulated genes involved in specific, significant canonical signaling pathways in acute stage monocytes relative to control subjects

To identify transcriptomic fingerprints of monocyte function, we performed bulk RNA-sequencing (RNA-seq) of CD14+ monocytes obtained directly from the circulation of CAP patients at the acute and recovery stages, as well as age and sex-matched control participants (Table 1). Principal component analysis of all detected transcripts (*n*=32,630) showed clear partitioning between controls and CAP patient monocytes during the acute stage (Fig. 3A). No clear cluster was observed for patient monocytes at recovery relative to controls. Compared to controls, we identified 1639 upregulated and 1299 downregulated transcripts in monocytes of CAP patients at the acute stage (adjusted *p*<0.01; fold expression ≥ +1.5 or ≤ −1.5) (Fig. 3B). Minimal transcriptional differences were detected in recovery monocytes relative to controls (13 upregulated and 1 downregulated gene), including complement lysis inhibitor, clusterin (*CLU*), as well as iron homeostasis genes *ALAS2*, *HBA1*, *HBB*, and *HBD* (Fig. 3C). Upregulated genes in monocytes during the acute stage were associated with various canonical signaling pathways related to cell metabolism, cytokine signaling, cell cycle, antimicrobial responses, phagocytosis, transcriptional regulation, and disease pathways (Fig. 3D). Genes involved in cholesterol biosynthesis (including *LBR*, *DHCR24*, *SC5D*, and *SQLE*), IL-10 signaling (containing *IL10*, *IL10RB*), complement system (with *C1QA*, *C1QB*, *C2*, and *CR1*) and cyclins/cell cycle regulation (including *CCNA1*, *CCNB1*, and *CDK1*) were amongst the most significantly upregulated pathways (Fig. 3D, E). The pathway analysis of downregulated genes during the acute stage of CAP revealed an association with mainly antigen presentation, e.g., *HLA-DOA*, *HLA-DPB1*, *HLA-DRA*, and *HLA-DMA*, cell activation, growth signaling, including B cell development, Th2 pathway, and IL-4 signaling, as well as a PD1/PDL1 pathway (Fig. 3D, E). Collectively, these data showed transcriptomes of circulating monocytes obtained from CAP patients during the acute stage were substantially altered relative to controls and primarily associated with cell metabolism, cytokine signalling, cell cycle, mobility, and antibacterial pathways. Those changes were concomitant with reduced expression of genes involved in antigen presentation. After 1 month, monocyte transcriptomes were largely similar to control participants, indicating resolution of the initial robust transcriptional response.

### Monocyte DNA methylation levels are partially modified in regulatory regions during the acute stage of pneumonia

**Fig. 4:**
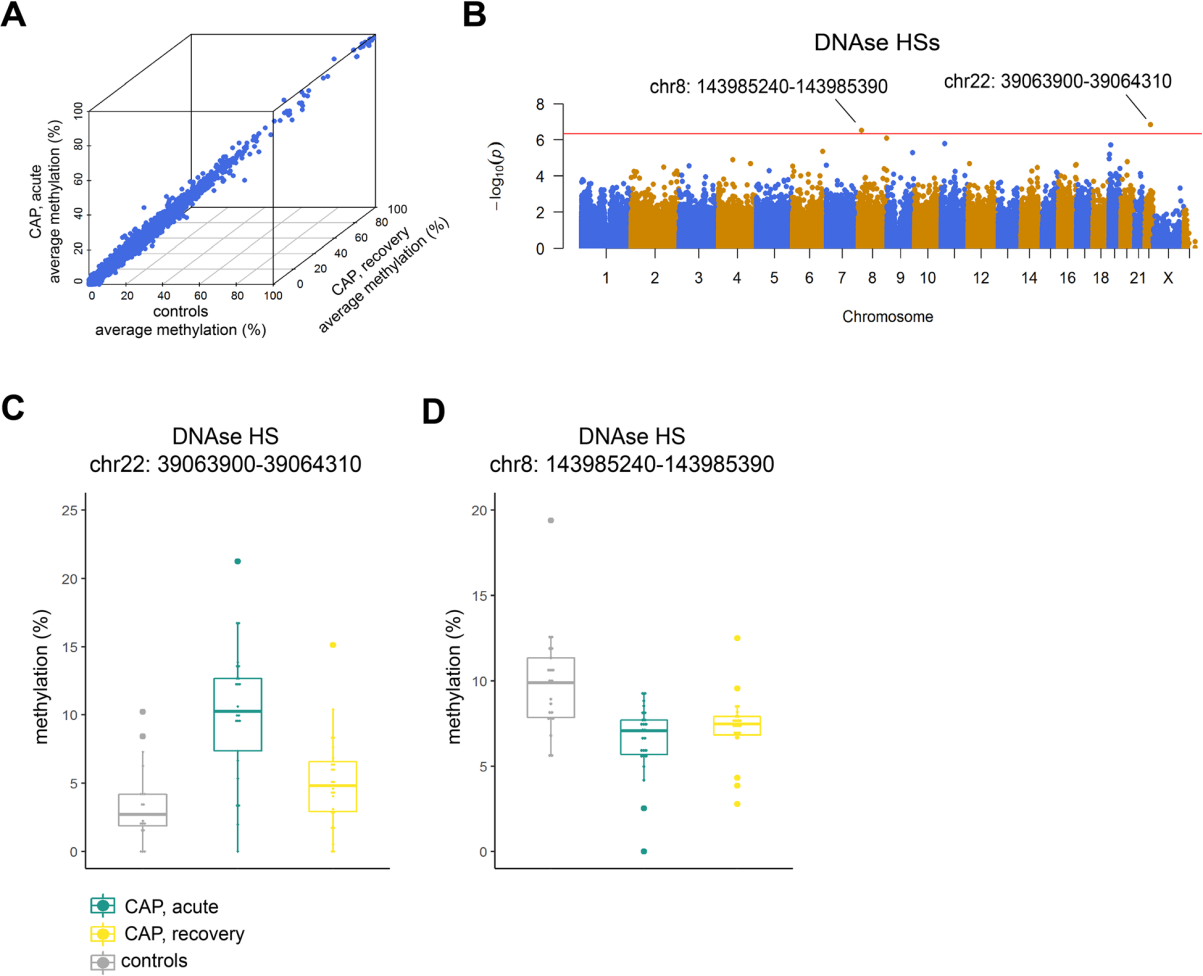
Monocyte DNA methylation assessed by reduced representation bisulfite sequencing in the acute and recovery stage of community-acquired pneumonia. **A** Three-dimensional scatter plot of methylation levels calculated in DNAse hypersensitive sites (DNAse-HS; 109,925 sites) of CAP patient monocytes obtained during the acute stage (*n* = 26), and from some of the same patients during the recovery stage (*n*=24), as well as age and sex-matched control subjects (*n* = 22). **B** Manhattan plot illustrating differentially methylated regions between CAP patient acute stage monocytes and controls. Horizontal red line denotes genome-wide significance threshold. **C** Boxplots of significantly altered DNAse HS methylation levels in CAP relative to controls

DNA methylation is an essential component of epigenetic phenomena that define cell identity and ultimately function. It occurs primarily in the context of symmetrical CG-dinucleotides and is understood to carry out divergent functions depending on the genomic region, for example gene promoters, exons, or repeated regions [10]. Here, we performed DNA methylation analysis at single-base resolution by reduced representation bisulfite sequencing (RRBS) [37, 38]. RRBS was done on circulating CD14+ monocytes obtained from a subset of CAP patients (acute, *n*=26; recovery, *n*=24) and control subjects (*n*=22), after matching for demographics and comorbidities (Additional file 3: Table S1). Overall, methylation levels were highest in the CG-dinucleotide context with no differences between study groups (Additional file 2: Fig. S2A). Summarizing CG-dinucleotides (>10x coverage) to specific regions of the GRCh38 genome assembly, and taking a 95% call-rate, resulted in 53,812 exons, 67,271 introns, 8329 promoters, 21,346 CpG islands (CGI), and 109,925 DNAse hypersensitive sites (HS) available for further analysis. Averaged methylation levels per genomic region were strongly similar between control subjects and CAP patients’ monocytes obtained during the acute disease stage, or after a 1-month recovery from the same patients (Fig. 4A and Additional file 2: Fig. S2B). To compare methylation levels, we used logit-transformed methylation levels to fit a multivariate linear model that included age, sex, and sequencing library pool as covariates. This approach identified two significantly altered DNAse HSs in CAP acute stage monocytes (relative to controls) located in chromosome (chr) 22 (39,063,900bp-39,064,310bp; DNAse-HS-Chr22) and chr8 (143,985,240bp-143,985,390bp; DNAse-HS-Chr8) (Fig. 4B). Higher methylation levels were determined for DNAse-HS-Chr22 in CAP acute stage monocytes; lower methylation levels were found for the chr8:143,985,240bp-143,985,390bp DNAse HS (Fig. 4C). No statistically significant differences were identified for CAP recovery monocytes relative to controls. DNAse-HS-Chr22 is located within a genomic region dense in C to U RNA-editing cytidine deaminases, apolipoprotein B mRNA editing enzymes (Additional file 2: Fig. S2C), and DNAse-HS-Chr8 is located in exon 4 of Poly(ADP-Ribose) Polymerase Family Member 10 (*PARP10*), a member of poly(ADP-ribose) polymerases that regulate chromatin architecture by adding ADP-ribose to histones [39] (Additional file 2: Fig. S2D). Expression patterns of apolipoprotein B mRNA editing enzymes and *PARP10* were not correlated to methylation levels of the respective DNAse HS (Additional file 2: Fig. S2E). Methylation levels at both DNAse HS regions are consistent with Blueprint epigenome signatures of CD14+ (classical) monocytes (Additional file 2: Fig. S3A, S3B) [40]. Based on the current genome build (hg38), DNAse-HS-chr22 harbors an ENCODE distal enhancer element annotated as EH38E2164281. To test for putative regulatory contacts in the genome, we downloaded and examined promoter capture Hi-C interactions specifically for monocytes and macrophages [30]. We found no sequence that matched the DNAse-HS-Chr22 genomic region; therefore, promoter targets could not be identified.

Next, we quantified 5-methylcytosine (5-mC) levels in monocytes obtained from a subset of CAP patients (*n*=21), captured during the acute disease stage, as well as control participants (*n*=16) by methylated DNA immunoprecipitation (MeDIP) [41]. The characteristics of CAP patients and controls are tabulated in Additional file 3: Table S2. Quantification of immunoprecipitated 5-mC DNA relative to total (input) DNA by quantitative PCR analysis showed elevated methylation levels at DNAse-HS-Chr22 in CAP patient monocytes relative to controls, which corroborated the levels detected in the RRBS experiment. However, a Wilcoxon rank sum test showed no statistically significant difference between CAP acute stage monocytes and control monocytes (*p*=0.059; Additional file 2: Fig. S4A). No differences were observed at DNAse-HS-Chr8 (Additional file 2: Fig. S4B). We also explored DNA methylation levels at DNAse-HS-Chr22 and DNAse-HS-Chr8 in an external, publicly available microarray study on DNA methylation levels in CD14+ monocytes of critically ill patients with sepsis due to pneumonia (*n*=4), and a subset of healthy (age, sex-matched) control subjects (*n*=6) (GSE138074) [29]. Beta values (indicative of methylation levels in beadchip technology) of probes located within DNAse-HS-Chr22 (1 probe) or DNAse-HS-Chr8 (3 probes) partly support our findings, particularly elevated methylation levels of the DNAse-HS-Chr22 probe in sepsis patients (Additional file 2: Fig. S4C, S4D). Altogether, our findings showed that DNA methylation levels of circulating CD14+ monocytes, obtained at the acute stage of CAP and after a 1-month recovery, were largely similar to control participants. DNA methylation in genomic regions harboring DNAse-HSs were partially altered in acute stage monocytes relative to controls.

### Multi-scale model of DNA methylation, RNA transcription, and variation in cytokine production

**Fig. 5:**
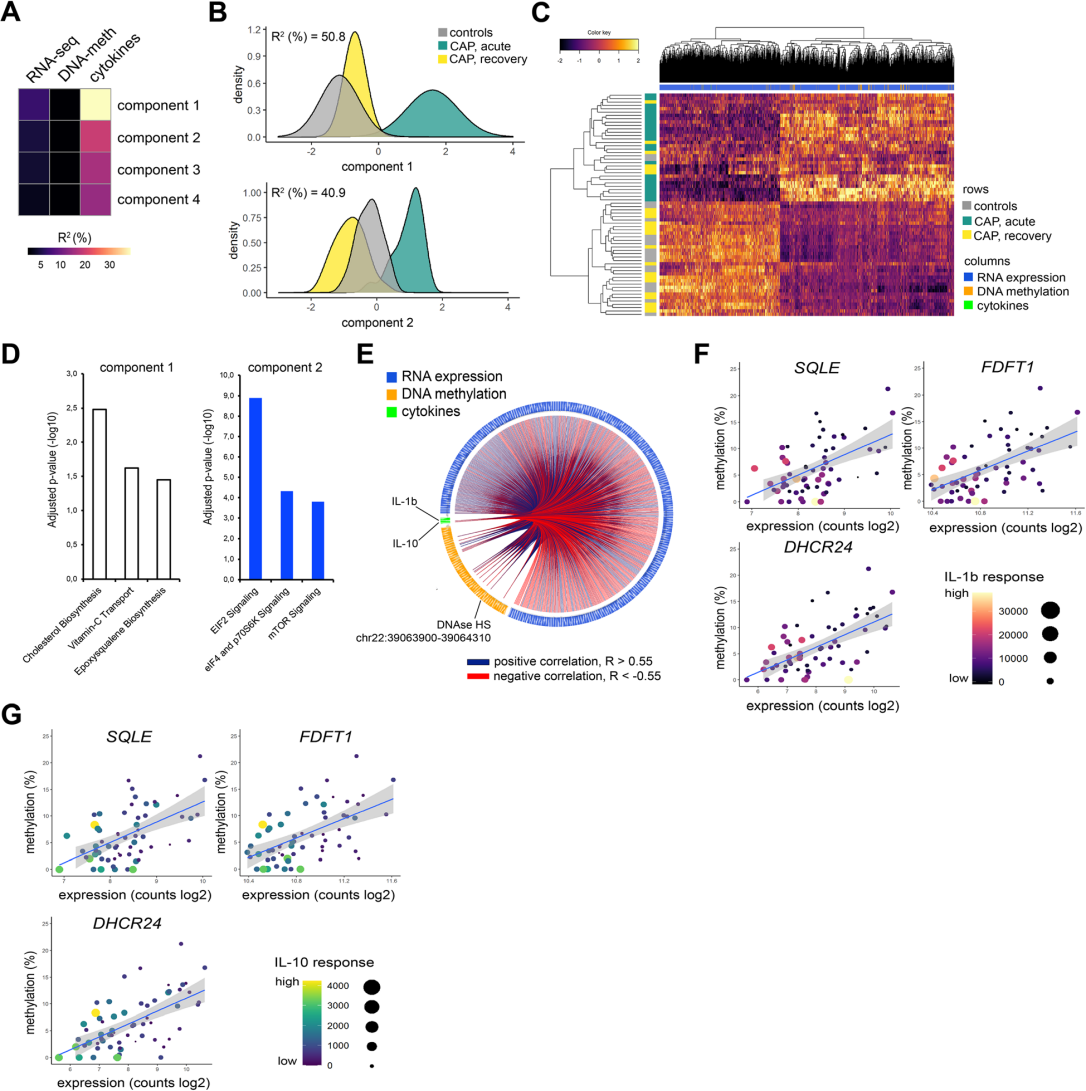
Multi-omics model of endotoxin tolerance in circulating monocytes obtained during the acute and recovery stage of community-acquired pneumonia. **A** Heatmap of percent variance explained (coefficient of determination, *R*^2^) per projection-to-latent-structure (PLS) component (1–4) for monocyte ex vivo cytokine production levels, DNAse hypersensitive site (DNAse-HS; 109,925 sites) DNA methylation levels and RNA transcriptomes (32,630 transcripts). **B** Density plots of percent explained variation in study groups per PLS component 1 and 2. **C** Clustered image heatmap of the most informative transcripts (*n*=492) and DNAse-HS methylation levels (*n*=92), taking loading vectors in the upper or lower 20% of the distribution in component 1, as well as ex vivo cytokine levels of TNF, IL-1β, IL-6, and IL-10. **D** Bar plots illustrating significantly enriched (multiple-test adjusted *p* < 0.05) Ingenuity canonical signaling pathways for PLS component 1 or 2. **E** Circular plot of tightly correlating (Pearson correlation cutoffs set at ≥ 0.55 or ≤ −0.55) ex vivo cytokine levels, RNA transcripts and DNAse-HS methylation levels in PLS component 1. **F**, **G**) Dot plots integrating percent methylation levels at DNAse-HS-Chr22, RNA expression levels of cholesterol biosynthesis genes *SQLE*, *DHCR24*, and *FDFT1*, as well as levels of IL-1B (**F**) and IL-10 (**G**) after ex vivo LPS exposure. Blue line denotes the line-of-best-fit and confidence intervals

To understand the relationship between DNAse HS methylation, RNA expression, and cytokine responses, we applied data integration analysis for biomarker discovery using latent components (DIABLO) [27]. DIABLO is a supervised framework to perform dimensionality reduction by applying projection to latent structure (PLS) models across all data modalities and feature selection using partial least squares discriminant analysis. We used RNA-seq (protein-coding and non-coding RNA; *n*=32,868), DNA methylation in DNAse HSs (*n*=109,925), and ex vivo cytokine production levels (TNF, IL-1β, IL-6, and IL-10), as data inputs to the model. For the latter, we calculated the change in cytokine levels after stimulation relative to medium (delta pg/mL) as a measure of cytokine inducibility. Computing the first 4 PLS components revealed the highest explained variance (*R*^2^) in PLS components 1 and 2 for cytokine responses (39.3% and 22.7%), RNA-seq (9.5% and 6.8%) and DNA methylation (2.4% and 2.6%) (Fig. 5A and Additional file 2: Fig. S5A, S5B), which also captured differences between study groups, with *R*^2^ equating to 50.8 and 40.9% for components 1 and 2, respectively (Fig. 5B). Focusing on the top two components, and selecting RNA-seq and DNA methylation features with loading vectors in the upper or lower 20% of the distribution (Additional file 2: Fig. S5C, S5D), identified DNA methylation and transcriptomic signatures for component 1 (492 RNA transcripts, 95 DNAse HSs) and component 2 (309 RNA transcripts, 91 DNAse HSs) (Fig. 5C and Additional file 2: Fig. S5E). The pathway analysis showed component 1 was associated with cholesterol biosynthesis pathway, whereas component 2 was enriched with EIF2 signaling (protein translation) genes (Fig. 5D). Therefore, DIABLO connected variation in monocyte responsiveness during pneumonia to changes in cholesterol biosynthesis, protein translation pathways, and DNAse HS methylation. Notably, in component 1, higher methylation levels at DNAse-HS-Chr22 were associated with elevated expression of key cholesterol biosynthesis genes that included *SQLE*, *FDFT1*, and *DHCR24*, concomitant with reduced production of IL-1β and IL-10 after LPS stimulation (Figs. 5F and G). Interestingly, enzymes involved in the early phases of cholesterol biosynthesis, including *HMGCR*, *HADHA*, and *MVK* were not associated with cytokine responses nor methylation levels at DNAse-HS-Chr22 (Additional file 2: Fig. S5F). Furthermore, reduced methylation in DNAse-HS-Chr22 was related to higher expression of genes involved in protein translation including *AARS2*, belonging to the family of aminoacyl-tRNA synthetases, and mitochondrial ribosomal protein S25 (*MRPS25*), as well as elevated cytokine responses (Additional file 2: Fig. S5G). Collectively, our observations showed diminished cytokine production of circulatory CD14+ monocytes during pneumonia reflects, at least in part, coordinated changes in DNA methylation at specific DNAse HSs, as well as genes involved in cholesterol biosynthesis (specifically down-stream reactions) and protein translation pathways.

## Discussion

In the current study, we adopted a systems-based multi-omics approach to unmask a DNA methylation and transcriptomic axis connected to the cytokine production capacities of circulating monocytes obtained from CAP patients. Patient monocytes sampled during the acute stage of the disease were reprogrammed to a state of immune tolerance, which was largely resolved after a 1-month recovery. Circulating acute stage monocytes displayed dramatic transcriptional changes with elevated genes involved in cell metabolism, mobility, cell cycle, cytokine signaling, and antimicrobial pathways, concomitant with reduced expression of genes involved in protein translation and antigen presentation. Those same monocytes exhibited largely unchanged DNA methylation levels, but specific regulatory regions of the genome, typically harbored in DNAse HSs, were partially altered. In particular, expression of genes involved in cholesterol biosynthesis was related to altered DNA methylation levels at specific DNAse HSs, which correlated with the production capacity of IL-1β and IL-10 by circulating monocytes.

Multi-omics approaches are emerging powerful tools to understand the flow of information from the cause of diseases to functional consequences, and relevant interactions between the different molecular strata [42]. It is evident that multiple factors and events contribute to infectious diseases, as well as potentially detrimental consequences such as host immunosuppression, which is the focus of the current report. The phenomenon of host immunosuppression has been suggested to confer a high risk of adverse outcomes to nosocomial infection [43, 44], exemplified by post-mortem studies showing death in sepsis mainly reflected unresolved opportunistic infections [44–46]. Various factors contributing to immunosuppression have been proposed, including apoptosis of lymphocytes [43, 47, 48] and changes in immune cell function and responses [49]. Conforming to the latter, the induction of endotoxin tolerance in monocytes and macrophages represents a potential mechanism of immunosuppression, wherein prolonged exposure to LPS leads to a state of cellular refractoriness to subsequent LPS exposure [50]. In addition, the effect of endotoxin tolerance on monocytes was not limited to a few pro-inflammatory genes, but also on a more genome-wide scale [51]. Evidence on in vitro stimulated monocytes or macrophages also points to epigenetic changes, in particular histone acetylation and methylation, which are understood to partially convey the effects of endotoxin tolerance [11, 16, 52]. Whether this phenomenon is regulated in vivo by changes to the DNA methylome of monocytes, and if it is fully resolved after recovery, are not well-understood.

In order to clarify this, we designed our split-sample study to compare monocytes obtained from patients diagnosed with CAP, as well as age and sex-matched control subjects. The same patients were sampled during the acute stage of the disease (on study inclusion), and after a 1-month follow-up (recovery stage). Our observations show that CAP patients’ monocytes sampled during the acute stage were impaired in pro-inflammatory cytokine release to ex vivo LPS challenge, indicating endotoxin tolerance. These findings are largely in line with previous reports in sepsis, particularly for TNF, IL-1β, and IL-6 [50]. Our observation on IL-10 production, which was also impaired in acute stage monocytes, contrasts previous reports showing either no effect or, at times, even enhanced production of IL-10 in endotoxin tolerance [50, 53]. The discordancy can be attributed to our experimental setup, designed specifically to evaluate circulating monocytes, thus avoiding the confounding aspects of monocyte-to-macrophage differentiation [54, 55], which is often overlooked. However, in vivo animal studies of IL-10 knock-out mice have demonstrated no effect of IL-10 deficiency on the induction of endotoxin tolerance [56]. Therefore, our study of non-septic CAP patient monocytes provides benchmark evidence that the phenomenon of endotoxin tolerance is not exclusive to sepsis and lends further weight to challenge the current paradigm that endotoxin tolerance is a pathophysiological mechanism which dampens pro-inflammatory mediators (e.g., TNF, IL-1β, IL-6) while activating anti-inflammatory factors such as IL-10. Furthermore, we observed that IL-6 production was still impaired after a 1-month follow-up, unlike TNF, IL-1β, and IL-10, which were relatively resolved, suggesting cytokine-specific footprints of endotoxin tolerance in circulating monocytes, subsequent to CAP, are more far-reaching than currently appreciated. Circulatory monocytes are known to have a short life span, with a recent elegant study in human endotoxemia and humanized animal experiments showing monocytes having a circulating lifespan of ~1 day [36]. The phenotypic differences observed in recovery monocytes of CAP patients cannot be explained by shifts in monocyte subsets, as classical, non-classical, and intermediate monocyte subsets were not different when compared to control participants. Therefore, we can only speculate that the sustained reduction in IL-6 production may be related to an as yet undiscovered mechanism of immune tolerance that leaves an indelible fingerprint of past infection in bone marrow myeloid progenitors. Recent studies have indeed shown that a stimuli, for example, a western-type diet in mice or bacillus Calmette-Guerin vaccination in humans, alter circulatory cell phenotypes that can last for months after the initial challenge by modifying hematopoiesis, and long-term reprogramming of bone marrow progenitor cells [57, 58]. Long-term reprogramming of innate immune cells is understood to be conveyed at multiple levels, including cell metabolism and epigenetics [7]. While our RRBS analysis did not uncover differences in DNA methylation between monocytes of CAP patients obtained after 1 month of study inclusion, relative to those of matched control participants, we cannot exclude the potential influence of other epigenetic features, for example, histone modifications, or cell metabolites on a long-term innate immune cell reprogramming. Therefore, the effect of lower respiratory tract infections on persistent reduction of IL-6 production capacities in circulatory monocytes via a long-term reprogramming of bone marrow myeloid progenitor cells certainly warrants further investigation.

Cellular phenotypes are regulated by a complex and dynamic interplay of various factors that include epigenetic changes such as DNA methylation [10]. Globally, the amount of variance in cytokine production explained by DNA methylation in our multi-omics model was small (5% cumulative PLS components 1 and 2; Fig. 5A). Moreover, direct comparison of monocyte DNA methylation levels of CAP patients and control participants showed largely similar patterns. These observations are in line with other (unrelated) studies on DNA methylation profiling of circulating CD14+ monocytes, showing predominantly similar patterns between cases and controls in, for example, Crohn’s disease [59], systemic lupus erythematosus [60], asthma [61], atherosclerosis [62], and sepsis [29]. This suggests that DNA methylation may not constitute a main feature of circulating CD14+ monocyte plasticity. However, DNA methylation changes have been associated to genes involved in specific pathways, including the IL-10 signaling pathway [29]. Genetic variants of a prototypical DNA methyltransferase encoded by *DNMT3A* were demonstrated to alter macrophage function in vitro, as well as the host response in a murine model of methicillin-resistant *Staphylococcus aureus* bacteremia [63]. Gain-of-function variants were associated with increased methylation and reduced expression of IL-10, which was related to a protective immune response and increased capacity for pathogen clearance. Consistent with this pattern, our findings showed that increased DNA methylation at DNAse-HS-Chr22 in CAP patient monocytes was associated with reduced production of IL-10 after exposure to LPS ex vivo.

By integrating different molecular strata, we unmasked a potentially essential link between DNA methylation and endotoxin tolerance via a transcriptomic signature attuned to cholesterol biosynthesis. Accumulation of mevalonate, produced during the process of cholesterol biosynthesis, was demonstrated to play an integral role in β-glucan induced trained immunity in human monocytes [15]. Furthermore, mevalonate also influenced, at least in part, the epigenetic landscape of monocytes that conveyed training. In the context of trained immunity, a process that conveys non-specific (long-term) memory in innate immunity [8], metabolites that accumulate downstream of the cholesterol biosynthesis pathway were not found to be essential [15]. Surprisingly, and in contrast, our findings demonstrate that genes involved in the production and handling of metabolites upstream in the cholesterol biosynthesis pathway, including mevalonate kinase (*MVK*), *HADHA*, and *HMGCR* were not associated with the induction of endotoxin tolerance. Our data showed that genes functioning in more downstream reactions of the cholesterol biosynthesis pathway, particularly the squalene enzyme encoded by *SQLE* and, further downstream, 24-dehydrocholesterol reductase (*DHCR24*) may be important mediators of endotoxin tolerance in human monocytes during the acute stage of CAP. In line with our observations, downstream metabolites of the cholesterol biosynthesis pathway were shown to affect chromatin remodeling mediated by the induction of LXRα [64, 65].

Our study has some limitations. First, since we employed a reduced representation approach to DNA methylation profiling, which permitted optimal coverage (>10x), we cannot exclude the possibility that other regions of the DNA methylome are involved in the induction of endotoxin tolerance due to CAP. Second, immune tolerance was ascertained using only bacterial endotoxin, albeit a well-established experimental model. Whether the DNA methylation and transcriptomic axis we identified is also relevant for other inducers of immune tolerance in circulating monocytes cannot be excluded. Third, since studies in similar cohorts have not been reported thus far, validation of our findings in other patient cohorts is lacking. In order to partly address this study limitation, we examined publicly available data on DNA methylation levels in CD14+ monocytes (measured by microarray) from critically ill patients with sepsis due to pneumonia (GSE138074) [29]. Notwithstanding dissimilarities in type of patients, sample size limitations and differences between technologies used for DNA methylation analysis, and patterns of DNA methylation at DNAse-HS-Chr22 and DNAse-HS-Chr8 in septic patient monocytes were partly in line with our observations in non-septic CAP patients. Fourth, using publicly available data on promoter capture Hi-C, we could not detect 3D chromatin interactions with DNAse-HS-Chr22 specifically in monocytes or macrophages [30]. This may be due to a number of reasons, including the choice of genome build, where we used the more recent human genome build GRCh38/hg38, as well as capture thresholds in public datasets. Therefore, identifying gene interactions with DNAse-HS-Chr22 (annotated as ENCODE distal enhancer element EH38E2164281) remains an open question.

## Conclusions

The cellular phenotype of circulating CD14+ monocytes obtained from (non-septic) CAP patients was attuned to a state of immune tolerance, which was not fully resolved after a 1-month recovery. By leveraging on the emerging concepts of integrative bioinformatics, we identified a DNA methylation and cholesterol biosynthesis axis related to cytokine production capacities of circulatory CD14+ monocytes. More observational and functional studies will be needed to tease out the mechanisms by which epigenetics regulates immune tolerance, and our data can provide an important resource to this aim. Our findings may constitute a promising avenue in developing treatments to potentially reverse the paralyzing effects of immunosuppression on host defense.

## Supplementary Information

**Additional file 1.** List of reagents, resources and computational methods.

**Additional file 2: Supplementary figures (Fig. S1 – S5)**. **Fig. S1**: Flow cytometry analysis of purified monocytes. **Fig. S2**: Reduced representation bisulfite sequencing analysis of DNA methylation in circulating monocytes during the acute and recovery stage of community-acquired pneumonia. **Fig. S3**: Genome view of significantly altered methylation levels in acute stage circulatory monocytes relative to controls. **Fig. S4**: Exploration of DNA methylation loci in a subset of CAP patients and controls, as well as in the public domain. **Fig. S5**: Multi-omics integration of ex vivo cytokine response to LPS exposure, DNA methylation levels and RNA expression profiles.

**Additional file 3: Supplementary tables (Table S1 and S2)**. Table S1: Baseline characteristics of community-acquired pneumonia patients and control participants for reduced representation bisulfite sequencing. Table S2: Baseline characteristics of community-acquired pneumonia patients and control participants for methylated DNA immunoprecipitation analysis

## Data Availability

The primary datasets generated during this study are available in the Gene Expression Omnibus of the National Center for Biotechnology Information with accession numbers GSE160329 (https://www.ncbi.nlm.nih.gov/geo/query/acc.cgi?acc=GSE160329) [66] and GSE159474 (https://www.ncbi.nlm.nih.gov/geo/query/acc.cgi?acc=GSE159474) [67]. The software and computational tools used for data analysis are indicated in the manuscript and Additional file 1.
