# Supplementary material for: An epigenetic and transcriptomic signature of immune tolerance in human monocytes through multi-omics integration
Source: Genome Med. 2021 Aug 16;13:131. doi: 10.1186/s13073-021-00948-1 (PMC8365568; doi:10.1186/s13073-021-00948-1)
Supplement: Supplementary file 1 — Additional file 1. List of reagents, resources and computational methods. [file 13073_2021_948_MOESM1_ESM.docx]

**An epigenetic and transcriptomic signature of immune tolerance in human monocytes through multi-omics integration**

Xanthe Brands, Bastiaan W. Haak, Augustijn M. Klarenbeek, Joe Butler, Fabrice Uhel, Wanhai Qin, Natasja A. Otto, Marja E. Jakobs, Daniël R. Faber, René Lutter, W. Joost Wiersinga, Tom van der Poll and Brendon P. Scicluna

**Supplementary methods**

**List of reagents**

| REAGENT or RESOURCE | SOURCE | IDENTIFIER |
| --- | --- | --- |
| **Antibodies** | | |
| anti-human CD14 antibody | BD Bioscience | Cat#555399 |
| magnetic beads coated with anti-CD14 antibodies | Miltenyi Biotech, San Jose, CA | Cat#130050201 |
| anti-human CD66b-FITC antibody | BD Bioscience, San Jose, CA, | Cat#555724 |
| anti-human CD3-FITC antibody | Ebioscience | Cat#12003842 |
| anti-human CD14-APC antibody | BD Bioscience, San Jose, CA | Cat#555399 |
| anti-human CD16-Alexa Fluor 700 antibody | BD Bioscience, San Jose, CA | Cat#560713 |
| anti-human CD56-PE CY7 antibody | BD Bioscience, San Jose, CA | Cat#560916 |
| **Bacterial and Virus Strains** | | |
| LPS- Escherichia coli, 100 ng/ml, Ultrapure | Invivogen | Cat#0111:B4 |
|  |  |  |
| **Biological Samples** |  |  |
| Heparin tubes | BD Bioscience, San Jose, CA | Cat#367874 |
| endotoxin-free bovine serum albumin | Divbio Science | Cat#AK8917-0100 |
| Pyrogen-free RPMI | GIBCO | Cat#31870-025 |
| Fetal Calf Serum | Bodinco | Cat#S-FBS-SA-025 |
| **Chemicals, Peptides, and Recombinant Proteins** | | |
| Ficoll-Paque PLUS | GE Healthcare Life science | Cat#17144002 |
| phosphate buffered saline | Fresenius Kabi | Cat#M090001/02 |
| sterile, endotoxin-free EDTA (500mM) | Thermo Fisher | Cat#AM9260G |
| Glutamax | Thermo Fisher | Cat#35050-038 |
| Pyruvate | Thermo Fisher | Cat#11360-039 |
| NaN_3_ | Merck Millipore | Cat#8223350100 |
| RNAprotect Cell reagent | Qiagen | Cat#76526 |
| Gentamycin | Lonza | Cat#17-519Z |
| **Critical Commercial Assays** | | |
| Human TNF-α, IL-1β, IL-6 and IL-10  Luminex multiplex assay | R&D Systems Inc. | Cat#LXSAHM-04 |
| BioPlex 200 | BioRad | Cat#X10010027401 |
| AllPrep DNA/RNA mini kit | Qiagen | Cat#80004 |
| Qubit RNA HS Assay Kit | ThermoFisher | Cat#Q38255 |
| Qubit dsDNA HS Assay Kit | ThermoFisher | Cat#Q38251 |
| Premium RRBS kit | Diagenode | Cat#C02030033 |
| Methylamp Methylated DNA Capture Kit | Epigentek | Cat#P-1015-48 |
| KAPA RNA Hyperprep with RiboErase | Roche | Cat#08098131702 |
| **Deposited Data** | | |
| RNA sequencing | GEO database | GSE160331 |
| Reduced representation bisulfite sequencing | GEO database | GSE159474 |
| **Software and Algorithms** | | |
| R | R Core Team 2014, Vienna, Austria | Version 3.5.1 |
| FACSDiva Software | BD biosciences | Version 8.0.1 |
| FastQC method | Babraham bioinform. | Version 0.11.5 |
| Python (Linux/UNIX) | Python | Version 2.7.12 |
| Trimmomatic | Bolger et al., 2014 | Version 0.36 |
| HISAT2 | Kim et al., 2015 | Version 2.1.0 |
| HTseq | Anders et al., 2015 | Version 0.6.1p1 |
| DESeq2 | Love et al., 2014 | Version 1.26.0 |
| BS-seeker2 | Guo et al., 2013 | Version 2.1.8 |
| Bowtie2 | Langmead and Salzberg, 2012 | Version 2.3.4.3 |
| CGmaptools | Guo et al., 2018 | Version 0.1.2 |
| Ingenuity Pathway analysis software | Qiagen Bioinformatics |  |
| mixOmics | Rohart et al., 2017 | Version 6.10.8 |
| limma | Smyth, 2005 | Version 3.42.2 |
| ggplot2 | Ginestet, 2011 | Version 3.3.0 |
| Deconvolution of Absolute Immune Signal  https://giannimonaco.shinyapps.io/ABIS/ | Monaco et.al., 2019 |  |
| Other | | |
| cell-repellent surface 48-well plate | Greiner Bio-One | Cat#677970 |
| MACS LS columns | Miltenyi Biotech | Cat#130-042-401 |
| Magnetic separator for MACS | Miltenyi Biotech | Cat#130-091-051 |
