# Supplementary material for: An epigenetic and transcriptomic signature of immune tolerance in human monocytes through multi-omics integration
Source: Genome Med. 2021 Aug 16;13:131. doi: 10.1186/s13073-021-00948-1 (PMC8365568; doi:10.1186/s13073-021-00948-1)
Supplement: Supplementary file 2 — Additional file 2: Supplementary figures (Fig. S1 – S5). Fig. S1: Flow cytometry analysis of purified monocytes. Fig. S2: Reduced representation bisulfite sequencing analysis of DNA methylation in circulating monocytes during the acute and recovery stage of community-acquired pneumonia. Fig. S3: Genome view of significantly altered methylation levels in acute stage circulatory monocytes relative to controls. Fig. S4: Exploration of DNA methylation loci in a subset of CAP patients and controls, as well as in the public domain. Fig. S5: Multi-omics integration of ex vivo cytokine response to LPS exposure, DNA methylation levels and RNA expression profiles. [file 13073_2021_948_MOESM2_ESM.docx]

**An epigenetic and transcriptomic signature of immune tolerance in human monocytes through multi-omics integration**

Xanthe Brands, Bastiaan W. Haak, Augustijn M. Klarenbeek, Joe Butler, Fabrice Uhel, Wanhai Qin, Natasja A. Otto, Marja E. Jakobs, Daniël R. Faber, René Lutter, W. Joost Wiersinga, Tom van der Poll and Brendon P. Scicluna

**Supplementary Figures**

**Fig. S1:** Flow cytometry analysis of purified monocytes.

**Fig. S2:** Reduced representation bisulfite sequencing analysis of DNA methylation in circulating monocytes during the acute and recovery stage of community-acquired pneumonia.

**Fig. S3:** Genome view of significantly altered methylation levels in acute stage circulatory monocytes relative to controls.

**Fig. S4**: Exploration of DNA methylation loci in a subset of CAP patients and controls, as well as in the public domain.

**Fig. S5:** Multi-omics integration of *ex vivo* cytokine response to LPS exposure, DNA methylation levels and RNA expression profiles.


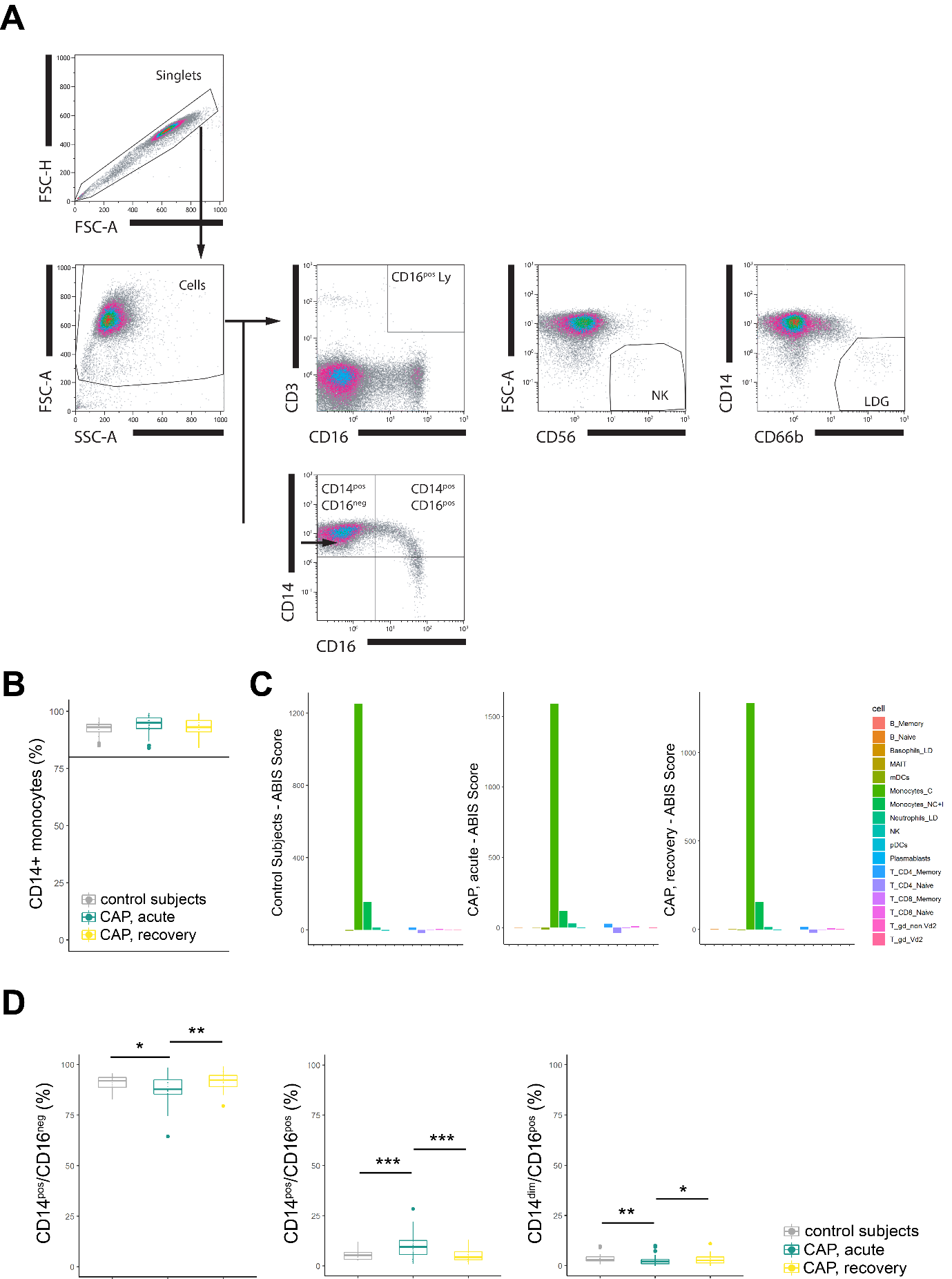


**Fig. S1: Flow cytometry analysis of purified monocytes.** (A) After exclusion of doublets and cell debris, CD14 positive (+) monocytes were gated as depicted and purity determined as percent CD14+ monocytes in the cell fraction. Purified monocytes were stained for CD3, CD14, CD16, CD56 and CD66b. After exclusion of residual CD3^pos^CD16^pos^ lymphocytes (CD16^pos^Ly), FSC^low^CD56^pos^ NK cells (NK), and CD14^neg^CD66b^pos^ low-density granulocytes (LDG), the three main monocyte subsets (CD14^pos^CD16^neg^, CD14^pos^CD16^pos^, and CD14^dim^CD16^pos^ monocytes) were gated as depicted. Gating strategy shown for one representative sample from a CAP patient. (B) Stripchart depicting the percent CD14+ monocytes in the cell fraction obtained from control subjects, community-acquired pneumonia (CAP) patients during the acute phase, and after one-month follow-up (recovery). Horizontal black line denotes 80% purity threshold (C) Barchart illustrating the absolute immune signal (ABIS) scores of CD14 monocyte transcriptomes obtained from control subjects, community-acquired pneumonia (CAP) patients during acute or recovery stages of the disease. (D) Boxplots depicting CD14 and CD16 cell-surface expression measured by FACS, characterizing classical (CD14^pos^/CD16^neg^), intermediate (CD14^pos^/CD16^pos^) or non-classical (CD14^dim^/CD16^pos^) monocyte subsets in experimental groups. Dunn’s post-hoc test p-values *<0.05, **<0.01 and ***<0.001.


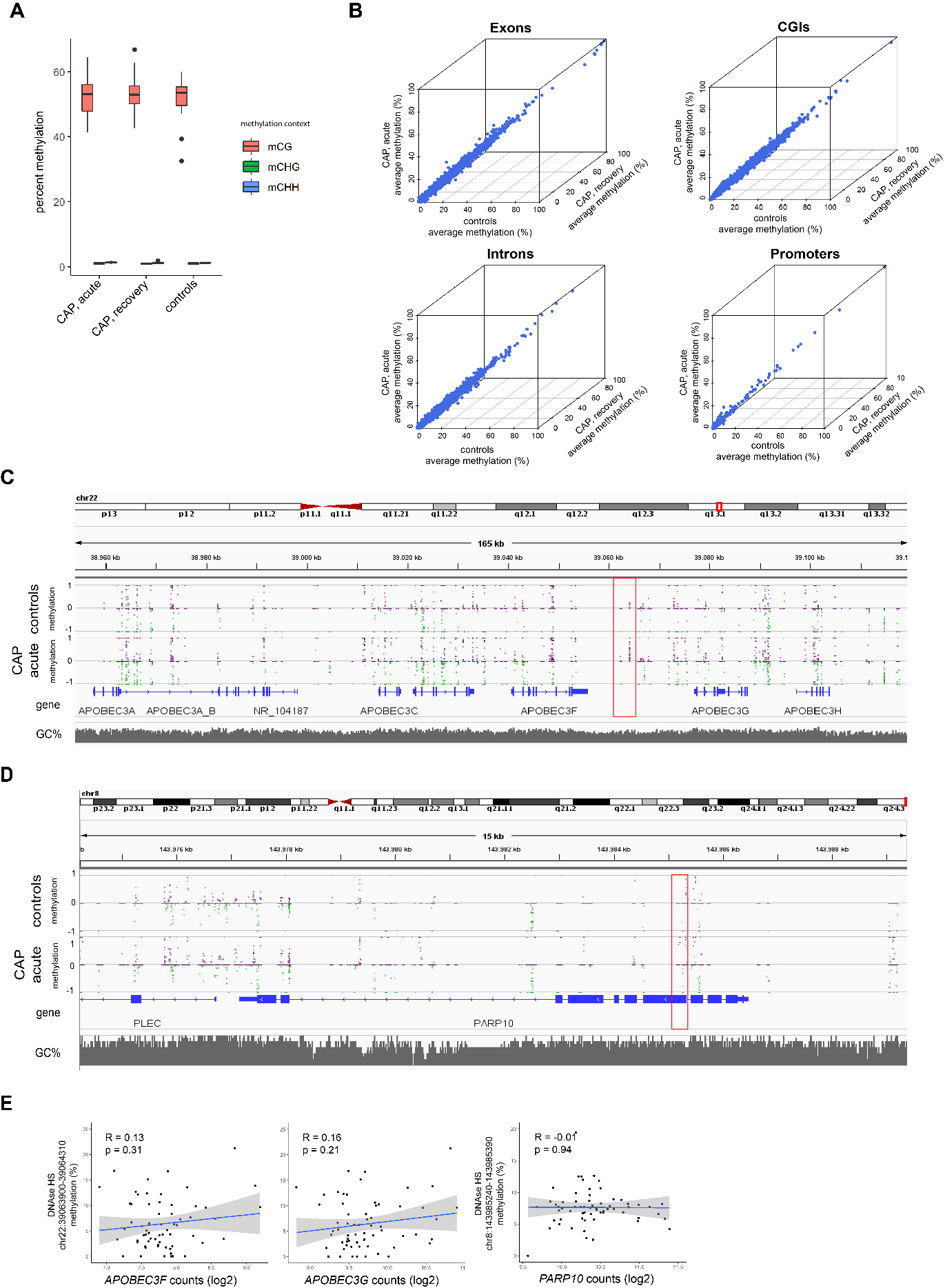


**Fig. S2: Reduced representation bisulfite sequencing analysis of DNA methylation in circulating monocytes during the acute and recovery stage of community-acquired pneumonia**. (A) Boxplot depicting overall methylation levels per dinucleotide and trinucleotide context across study groups. (B) Three-dimensional scatter plot of methylation levels calculated in exons (53,812 sites), introns (67,271 sites), CpG islands (CGIs; 21,346 sites) and promoters (8329 sites) of CAP patient monocytes obtained during the acute stage (on study inclusion, n = 26), and from the some of the same patients during the recovery stage (after one month follow-up, n = 24), as well as age and sex-matched control subjects (n = 22). (C and D) Integrative genome view screenshot of significantly altered DNAse hypersensistive sites on chromosomes 22 (C) and 8 (D). (E) Scatter plots of DNA methylation and RNA expression levels of genes proximal to methylation site. R, Pearson correlation.


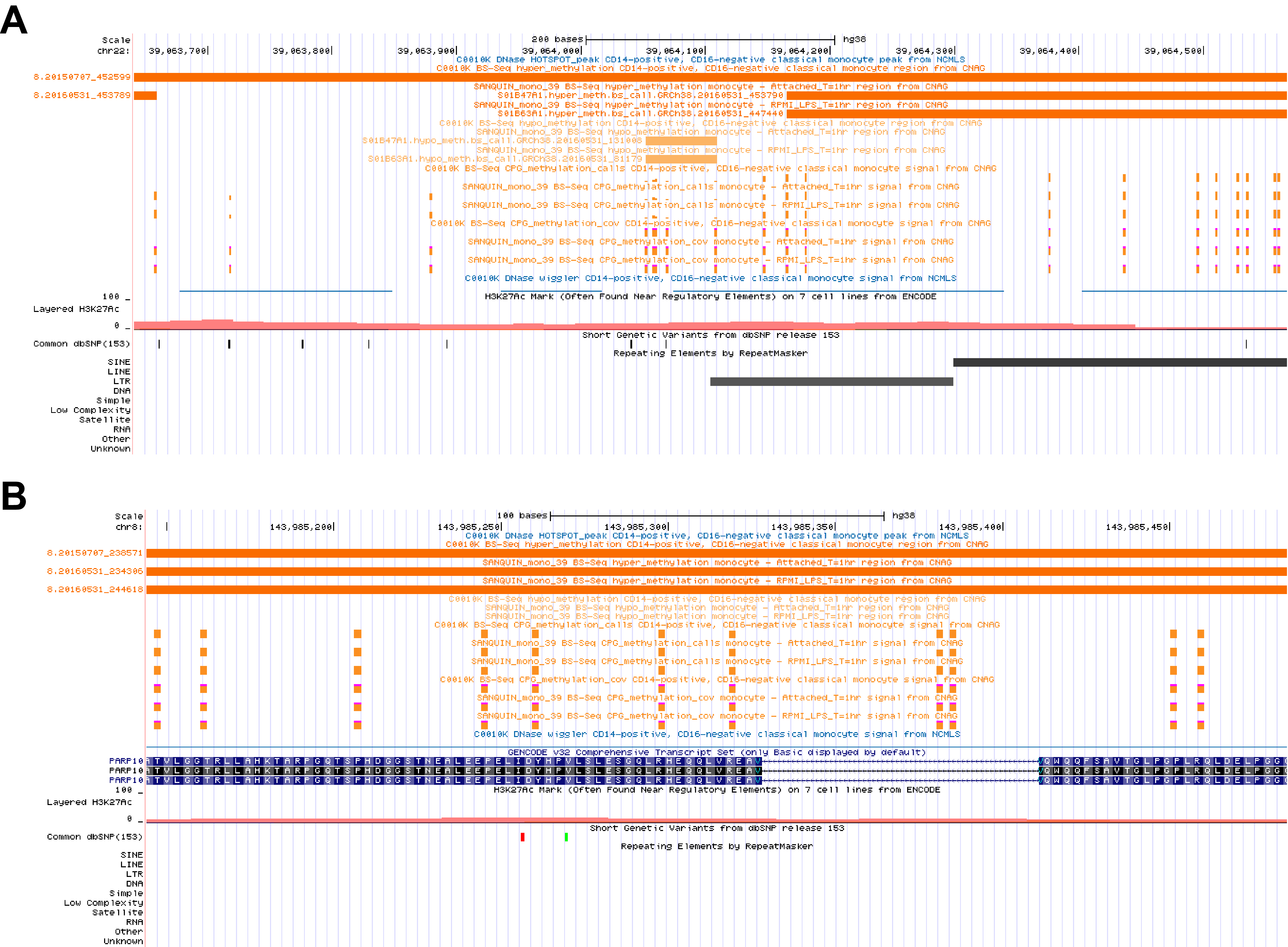


**Fig. S3: Genome view of significantly altered methylation levels in acute stage circulatory monocytes relative to controls**. University of California Santa Cruz (UCSC) genome view (build GRCh38) of DNAse hypersensitive sites on chromosomes 22 (A) and 8 (B) including Blueprint epigenome DNA methylation tracks for healthy human monocytes.

**
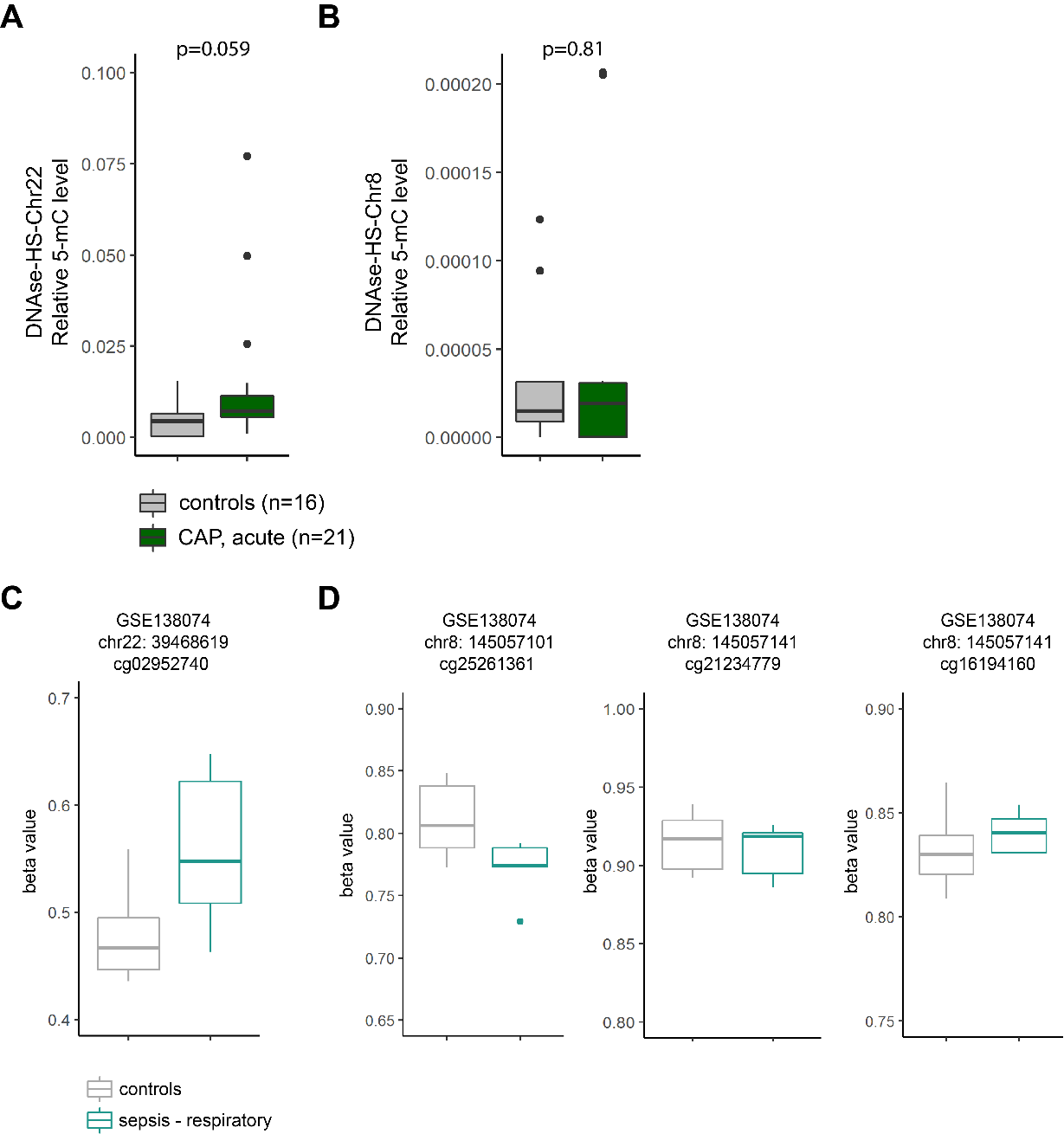
**

**Fig. S4**: **Exploration of DNA methylation loci in a subset of CAP patients and controls, as well as in the public domain.** (A and B) Boxplots illustrating methylated DNA immunoprecipitation (MeDIP) results of CAP patient monocytes obtained during the acute disease stage (n=21) and control participants (n=16). Quantitative PCR of 5-methylcytosine (5-mC) levels in immunoprecipitated DNA relative to input DNA at (A) DNAse-HS-Chr22, and (B) DNAse-HS-Chr8. P-values denote Wilcoxon rank sum test probabilities. (C and D) Boxplots depicting β values of Infinium MethylationEPIC BeadChip data on monocytes from patients with sepsis due to “respiratory” diagnosis (n=4) and healthy controls (n=6) available in GSE138074. Probe selection was based on the GRCh37 genome build that overlapped with DNAse-HS-Chr22 (A) and DNAse-HS-Chr8 (B).


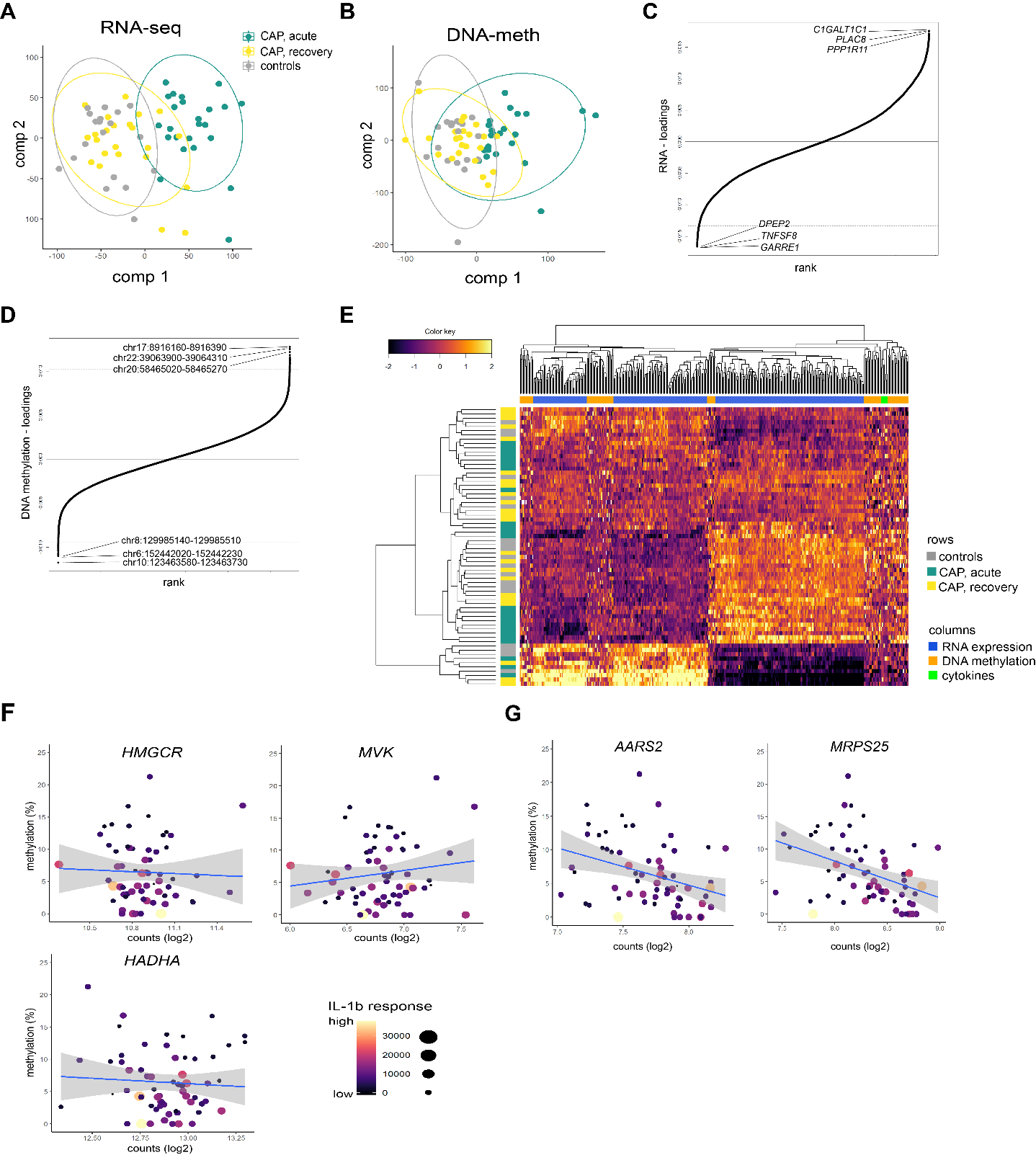


**Fig. S5: Multi-omics integration of *ex vivo* cytokine response to LPS exposure, DNA methylation levels and RNA expression profiles.** (A and B) Dot plots of RNA expression levels (A) and DNAse hypersensitive site methylation (B) in circulating monocytes of CAP patients obtained during the acute stage (n = 27), and from some of the same patients as at recovery (one month follow-up, n = 24), as well as control subjects (n = 22) considering projection-to-latent-structure (PLS) components 1 and 2. (C and D) Distribution of loading vectors for PLS component 1 of RNA expression (C) and DNAse HS methylation (D). (E) Clustered image heatmap of the most informative transcripts (n=309) and DNAse-HS methylation levels (n=91) with loading vectors in the upper or lower 20% of the distribution in component 2, as well as ex vivo cytokine levels of TNF, IL-1β, IL-6 and IL-10. (F) Dot plots integrating percent methylation levels at DNAse-HS-Chr22, RNA expression levels of up-stream cholesterol biosynthesis genes MVK, HADHA and HMGCR, as well as levels of IL-1β after ex vivo LPS exposure. (G) Dot plots integrating percent methylation levels at DNAse-HS-Chr22, RNA expression levels of protein translation genes (AARS2 and MRPS25) and ex vivo IL-1β levels. Blue line denotes the line of best fit and confidence intervals.
