# Supplementary material for: An epigenetic and transcriptomic signature of immune tolerance in human monocytes through multi-omics integration
Source: Genome Med. 2021 Aug 16;13:131. doi: 10.1186/s13073-021-00948-1 (PMC8365568; doi:10.1186/s13073-021-00948-1)
Supplement: Supplementary file 3 — Additional file 3: Supplementary tables (Table S1 and S2). Table S1: Baseline characteristics of community-acquired pneumonia patients and control participants for reduced representation bisulfite sequencing. Table S2: Baseline characteristics of community-acquired pneumonia patients and control participants for methylated DNA immunoprecipitation analysis [file 13073_2021_948_MOESM3_ESM.docx]

**An epigenetic and transcriptomic signature of immune tolerance in human monocytes through multi-omics integration**

Xanthe Brands, Bastiaan W. Haak, Augustijn M. Klarenbeek, Joe Butler, Fabrice Uhel, Wanhai Qin, Natasja A. Otto, Marja E. Jakobs, Daniël R. Faber, René Lutter, W. Joost Wiersinga, Tom van der Poll and Brendon P. Scicluna

**Supplementary Tables**

**Table S1:** Baseline characteristics of community-acquired pneumonia patients and control participants for reduced representation bisulfite sequencing

**Table S2:** Baseline characteristics of community-acquired pneumonia patients and control participants for methylated DNA immunoprecipitation analysis

**Table S1:** Baseline characteristics of community-acquired pneumonia patients and control participants for reduced representation bisulfite sequencing

|  | **CAP patients** | **Control subjects** | ***P*-value** |
| --- | --- | --- | --- |
| Size, n | 26 | 22 |  |
| **Demographics** |  |  |  |
| Age, yr, mean (SD) | 70.31 (11.90) | 70.23 (8.44) | 0.979^‡^ |
| Sex, male, n (%) | 13 (50.0) | 14 (63.6) | 0.511^†^ |
| Ethnicity, Caucasian, n (%) | 22 (84.6) | 21 (95.5) | 0.453^†^ |
| Body Mass Index, median [Q1, Q3] | 26.36 [25.01, 28.03] | 27.73 [26.04, 31.46] | 0.257^#^ |
| **Chronic comorbidities, n (%)** |  |  |  |
| COPD | 9 (34.6) | 3 (13.6) | 0.181^†^ |
| Cardiovascular disease | 21 (80.8) | 14 (63.6) | 0.315^†^ |
| Diabetes | 5 (19.2) | 2 (9.1) | 0.561^†^ |
| Malignancy | 7 (26.9) | 5 (22.7) | >0.99^†^ |
| Chronic renal disease | 1 (3.8) | 1 (4.5) | >0.99^†^ |
| Immunosuppression | 0 (0.0) | 2 (9.1) | 0.398^†^ |
| **Disease severity, median [Q1, Q3]** |  |  |  |
| PSI score | 4.00 [3.00, 4.00] | - |  |
| SOFA score | 0.50 [0.00, 2.00] | - |  |
| **Causal pathogen, n (%)** |  |  |  |
| *Streptococcus pneumoniae* | 2 (1.69) | - |  |
| *Haemophilus influenzae* | 1 (0.85) | - |  |
| *Staphylococcus aureus* | 2 (1.69) | - |  |
| Influenza A virus | 0 (0) | - |  |
| Influenza B virus | 0 (0) | - |  |
| Rhinovirus | 0 (0) | - |  |
| Coronavirus | 0 (0) | - |  |
| Respiratory syncytial virus | 1 (0.85) | - |  |
| Human metapneumovirus | 0 (0) | - |  |
| Parainfluenza virus 1-4 | 1 (0.85) | - |  |
| No causative pathogen found | 19 (16.1) | - |  |
| **Outcome** |  |  |  |
| ICU admission, n (%) | 2 (7.7) | ***-*** |  |
| Hospital LoS, days, median [Q1, Q3] | 5.00 [4.00, 7.00] | - |  |
| Hospital mortality, n (%) | 0 (0.0) | - |  |
| Day 28 mortality, n (%) | 0 (0.0) | - |  |

Definition of abbreviations: CAP = community-acquired pneumonia; COPD = chronic obstructive pulmonary disease; PSI = Pneumonia Severity Index; SOFA = Sequential Organ Failure Assessment; ICU = intensive care unit; LoS = length of stay; SD = standard deviation of the mean. * Immunosuppression was defined by use of methotrexate or prednisone, and/or positive human immunodeficiency virus.

^#^ Mann-Whitney U test; ^†^ Chi-square test with Yates' continuity correction; ^‡^ Student t-test.

**Table S2:** Baseline characteristics of community-acquired pneumonia patients and control participants for methylated DNA immunoprecipitation analysis

|  | **CAP patients** | **Control subjects** | ***P*-value** |
| --- | --- | --- | --- |
| Size, n | 21 | 16 |  |
| **Demographics** |  |  |  |
| Age, yr, mean (SD) | 71.00 (13.25) | 71.00 (8.89) | >0.99^‡^ |
| Sex, male, n (%) | 12 (57.1) | 9 (56.2) | >0.99^†^ |
| Ethnicity, Caucasian, n (%) | 14 (66.7) | 12 (75.0) | 0.852^†^ |
| Body Mass Index, median [Q1, Q3] | 24.69 [20.61, 27.20] | 26.84 [25.41, 28.52] | 0.059^#^ |
| **Chronic comorbidities, n (%)** |  |  |  |
| COPD | 6 (28.6) | 0 (0.0) | 0.059^†^ |
| Cardiovascular disease | 15 (71.4) | 12 (75.0) | >0.99^†^ |
| Diabetes | 9 (42.9) | 1 (6.2) | 0.035^†^ |
| Malignancy | 5 (23.8) | 5 (31.2) | 0.896^†^ |
| Chronic renal disease | 3 (14.3) | 2 (12.5) | >0.99^†^ |
| Immunosuppression | 6 (28.6) | 2 (12.5) | 0.439^†^ |
| **Disease severity, median [Q1, Q3]** |  |  |  |
| PSI score | 4.00 [3.00, 4.00] | - |  |
| SOFA score | 0.00 [0.00, 2.00] | - |  |
| **Causal pathogen, n (%)** |  |  |  |
| *Streptococcus pneumoniae* | 2 (1.69) | - |  |
| *Haemophilus influenzae* | 1 (0.85) | - |  |
| *Staphylococcus aureus* | 0 (0.0) | - |  |
| Influenza A virus | 1 (0.85) | - |  |
| Influenza B virus | 2 (1.69) | - |  |
| Rhinovirus | 1 (0.85) | - |  |
| Coronavirus | 0 (0) | - |  |
| Respiratory syncytial virus | 0 (0) | - |  |
| Human metapneumovirus | 1 (0.85) | - |  |
| Parainfluenza virus 1-4 | 1 (0.85) | - |  |
| No causative pathogen found | 14 (11.86) | - |  |
| **Outcome** |  |  |  |
| ICU admission, n (%) | 0 (0.0) | ***-*** |  |
| Hospital LoS, days, median [Q1, Q3] | 6.00 [3.00, 10.00] | - |  |
| Hospital mortality, n (%) | 0 (0.0) | - |  |
| Day 28 mortality, n (%) | 2 (11.1) | - |  |

Definition of abbreviations: CAP = community-acquired pneumonia; COPD = chronic obstructive pulmonary disease; PSI = Pneumonia Severity Index; SOFA = Sequential Organ Failure Assessment; ICU = intensive care unit; LoS = length of stay; SD = standard deviation of the mean. * Immunosuppression was defined by use of methotrexate or prednisone, and/or positive human immunodeficiency virus.

^#^ Mann-Whitney U test; ^†^ Chi-square test with Yates' continuity correction; ^‡^ Student t-test.
